# *Candida albicans* repetitive elements display epigenetic diversity and plasticity

**DOI:** 10.1038/srep22989

**Published:** 2016-03-14

**Authors:** Verónica Freire-Benéitez, R. Jordan Price, Daniel Tarrant, Judith Berman, Alessia Buscaino

**Affiliations:** 1 University of Kent, School of Biosciences Canterbury Kent, CT2 7NJ. UK; 2 Department of Microbiology and Biotechnology, George S. Wise Faculty of Life Sciences, Tel Aviv University, Ramat Aviv, 69978, Israel

## Abstract

Transcriptionally silent heterochromatin is associated with repetitive DNA. It is poorly understood whether and how heterochromatin differs between different organisms and whether its structure can be remodelled in response to environmental signals. Here, we address this question by analysing the chromatin state associated with DNA repeats in the human fungal pathogen *Candida albicans*. Our analyses indicate that, contrary to model systems, each type of repetitive element is assembled into a distinct chromatin state. Classical Sir2-dependent hypoacetylated and hypomethylated chromatin is associated with the rDNA locus while telomeric regions are assembled into a weak heterochromatin that is only mildly hypoacetylated and hypomethylated. Major Repeat Sequences, a class of tandem repeats, are assembled into an intermediate chromatin state bearing features of both euchromatin and heterochromatin. Marker gene silencing assays and genome-wide RNA sequencing reveals that *C. albicans* heterochromatin represses expression of repeat-associated coding and non-coding RNAs. We find that telomeric heterochromatin is dynamic and remodelled upon an environmental change. Weak heterochromatin is associated with telomeres at 30 °C, while robust heterochromatin is assembled over these regions at 39 °C, a temperature mimicking moderate fever in the host. Thus in *C. albicans*, differential chromatin states controls gene expression and epigenetic plasticity is linked to adaptation.

Large blocks of DNA repeats are commonly clustered at rDNA loci, telomeres and centromeres and are assembled into heterochromatin. Heterochromatic regions impose a transcriptionally repressive environment that can propagate over long distances (up to 50 kb) stochastically silencing native genes as well as reporter genes inserted at these regions independently of the underlying DNA sequence^1^,^2^,^3^,^4^. Transcriptionally repressive heterochromatin is distinguishable from transcriptionally active euchromatin by several epigenetic features where heterochromatin is characterised by nucleosomes that are hypoacetylated, hypomethylated on lysine 4 of histone H3 (H3K4) and methylated on lysine 9 of histone H3 (H3K9)^5^,^6^. Histone modifiers control the transcriptionally repressive state of heterochromatin regions via chromatin modifications. For example, the conserved histone deacetylase Sir2 controls the hypoacetylated state of heterochromatic regions while the histone methyltransferase Su(var)3–9 specifically methylates H3K9^1^,^2^,^7^,^8^,^9^,^10^,^11^. Heterochromatin has the ability to propagate thanks to specific silencing complexes. For example, in *Saccharomyces cerevisiae*, assembly of subtelomeric heterochromatin is mediated by the Sir silencing complex formed by Sir2, Sir3 and Sir4^1^. Likewise, heterochromatin assembly at the rDNA locus is dependent on the RENT (REgulator of Nucleolar silencing and Telophase) complex formed by Sir2, Net1 and Cdc14^12^,^13^,^14^. Heterochromatin modulation could be particularly important for organisms, such as microbial pathogens, that have to adapt rapidly to different environments. This is because heterochromatin can modulate gene expression without changes in DNA sequence. However, very little is known about heterochromatin-mediated transcriptional regulation in this group of organisms. Here we address this question by investigating the chromatin states associated with DNA repeats in the most common human fungal pathogen: *Candida albicans*. *C. albicans* normally lives as a commensal in humans but it can become virulent causing systemic life-threatening infections with mortality rates of up to 50%^15^. Upon environmental changes, *C. albicans* undergoes major morphological and genomic changes that can promote adaptation and survival^4^,^16^. It is unknown whether the chromatin structure of the *C. albicans* genome is also plastic and capable of remodeling upon environmental changes.

The *C. albicans* genome has 8 diploid chromosomes and contains 3 major classes of large blocks of repetitive DNA: the *rDNA* locus, Major Repeat Sequences (*MRS*) and telomeres^17^,^18^ (Fig. S1A). The *rDNA* locus consists of a tandem array of a ~12 kb unit repeated 50 to 200 times on chromosome R. Each unit contains the two highly conserved 35 S and the 5 S rRNA genes that are separated by two Non-Transcribed Regions (NTS1 and NTS2), whose sequences are not conserved across eukaryotes (Fig. S1B)^17^,^18^. In other organisms, while the 35S and 5S *rDNA* genes are highly expressed, the NTS1 and NTS2 regions are assembled in transcriptionally silent heterochromatin^12^,^13^,^14^. At these locations, heterochromatin represses transcription of non coding RNAs promoting stability of the rDNA loci^19^,^20^. The *MRS* loci are long tracts (10–100 kb) of nested DNA repeats found on 7 of the 8 *C. albicans* chromosomes^18^,^21^. These repetitive domains are formed by large tandem arrays of 2.1 kb RPS unit flanked by non-repetitive HOK and RBP-2 elements (Fig. S1C). In addition, a RBP-2 element, but not an intact MRS, is found on chromosome 3^22^. Given their highly repetitive nature, MRS repeats are expected to be ideal substrates for heterochromatin assembly. *C. albicans* telomeres are composed of a terminal region composed of tandemly repeating 23 bp units and subtelomeric regions (Fig. S1D)^23^. Due to their size and repetitive nature, the sequences of subtelomeric regions remain poorly characterised^18^.

DNA repeats are central to *C. albicans* genome plasticity and pathogenicity as they play key roles in the regulation of genome organisation and structure in the host^16^. However, the chromatin state of these DNA elements is unknown. The *C. albicans* epigenome, like the *S. cerevisae* epigenome, most probably lacks H3K9 methylation given that a Su(var) 3–9 orthologue cannot be identified in either *C. albicans* or *S. cerevisiae.* In contrast, the *C. albicans* genome encodes for two putative NAD-dependent histone deacetylases that resemble Sir2 (*orf19.1992* and *orf19.4762*)^24^. While it is possible to identify orthologues for RENT complex components, components of the Sir silencing complex are not apparent. Therefore, while heterochromatin might associate with the rDNA locus, it is possible that *C. albicans* telomeres lack heterochromatin as it has been recently shown for the yeast *Clavispora lusitaniae*^11^.

In this study, we investigated the chromatin states associated with *C. albicans* repetitive elements under different environmental conditions. We found that the different types of repetitive elements are assembled in distinct chromatin states. Classical hypoacetylated heterochromatin is associated with the non-transcribed region of the rDNA locus. The histone deacetylase Sir2 (*orf 19.1992*) is required to maintain this repressive epigenetic state via hypoacetylation of Lysine 9 of Histone H3. Heterochromatin associated with these regions represses non-coding RNA transcription. In contrast, MRS repeats are assembled into a transcriptionally permissive chromatin state bearing both heterochromatic and euchromatic histone marks. Finally, we find that, despite the apparent absence of the Sir silencing complex, telomeric regions are assembled into a Sir2-dependent hypoacetylated and hypomethylated heterochromatin. This chromatin state silences expression of endogenous transcripts as well as inserted marker genes. Telomeric heterochromatin is plastic and affected by environmental conditions with heterochromatin being more robust at higher temperature (39 °C) than at lower temperature (30 °C). Thus, the epigenetic state associated with telomeric repeats switches in response to an environmental change that are linked to *C. albicans* virulence and pathogenicity.

## Results

### Transcriptional silencing at the *C. albicans* rDNA locus

**Figure 1:**
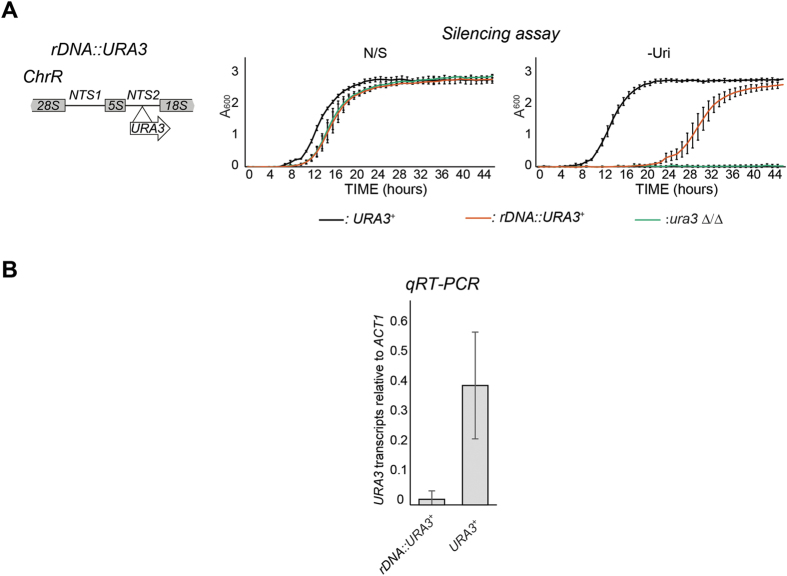
Transcriptional silencing at the *C. albicans* rDNA locus. (**A**) *Left panel*: Schematic of *rDNA::URA3*^+^ reporter strain*. Right panel*: silencing assay of the *rDNA::URA3*^+^ reporter strain. Ura^+^ (*URA3/URA3)* and Ura^−^ (*ura3*Δ*/ura3*Δ) strains were included as controls. (**B**) *qRT-PCR* analyses to measure *URA3*^+^ transcript levels of the *rDNA::URA3*^+^ reporter strain relative to actin transcript levels (*ACT1*). Error bars in each panel: Standard deviation (SD) of three biological replicates.

Heterochromatin assembled onto repetitive DNA represses the transcription of marker genes inserted in their proximity^25^,^26^,^27^,^28^. To assess whether transcriptionally silent chromatin exists in *C. albicans*, the *URA3*^+^ marker gene was integrated into the NTS2 region of the *rDNA* locus (*rDNA::URA3*^+^) (Fig. 1A). We investigated whether *URA3*^+^ was transcriptionally silenced when present at this locus, by growing strains in non-selective (N/S) medium and in medium lacking uridine (−Uri) in which only cells expressing sufficient Ura3 protein are able to grow. Silencing of *URA3*^+^ is expected to result in slower growth in –Uri medium compared to N/S. The *rDNA::URA3*^+^ strain displayed a reduced growth rate in selective medium and it is, therefore, silenced (Fig. 1A). Consistent with the growth assays the level of *URA3* mRNA levels at the *rDNA* locus were significantly lower (20 fold) for *rDNA::URA3*^+^ than for a *URA3*^+^ gene expressed from its own euchromatic locus, as determined by quantitative reverse transcriptase analysis (*qRT-PCR*) (Fig. 1B). Therefore, in *C. albicans*, the non-transcribed region of the rDNA locus is assembled into a transcriptionally repressed state that is normally associated with repetitive heterochromatic regions.

### Sir2-dependent heterochromatin at the rDNA locus

**Figure 2:**
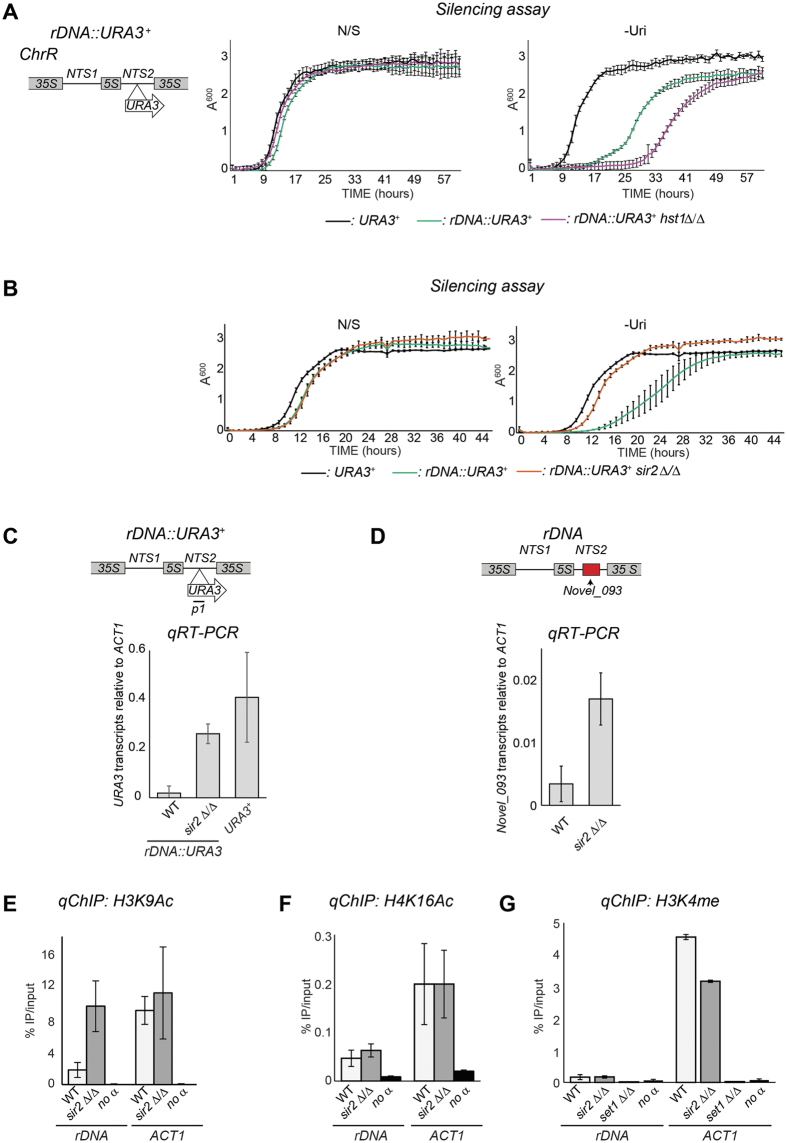
Sir2-dependent heterochromatin at the rDNA locus. (**A**) *Left panel*: Schematic of *rDNA::URA3*^+^ reporter strain. *Right panel*: Silencing assay of the *rDNA::URA3*^+^ reporter strain in WT and *hst1* Δ*/*Δ isolates. A URA^+^ (*URA3*^+^) strain was included as a control. (**B**) Silencing assay of the *rDNA::URA3*^+^ reporter strain WT and *sir2* Δ*/*Δ isolates. A URA^+^ (*URA3*^+^) strain was included as a control. (**C**) *qRT-PCR* analyses to measure *URA3*^+^ transcript levels relative to *ACT1* transcript levels in *rDNA::URA3*^+^ WT and *sir2* Δ*/*Δ isolates. A *URA3*^+^ strain is included as a control (**D**) *qRT-PCR* analyses to measure levels of the rDNA non-coding RNA (*Novel_Chr3_093*) relative to *ACT1* in WT and *sir2* Δ*/*Δ isolates. (**E,F**) *qChIP* to detect H3K9Ac, H4K16Ac levels associated with the *rDNA* locus and *ACT1* in WT and *sir2* Δ*/*Δ isolates and (**G**) H3K4me2 levels associated with the *rDNA* locus and *ACT1* in WT, *sir2* Δ*/*Δ and *set1* Δ*/*Δ isolates. Error bars in each panel: Standard deviation (SD) of three biological replicates.

The ability of *C. albicans* rDNA repeats to cause transcriptional silencing suggests that the rDNA NTS regions are assembled into heterochromatin. The histone deacetylase Sir2 is a key regulator of heterochromatin in all organisms studied^1^,^29^. BLAST analyses reveal that the *C. albicans* genome contains 5 genes encoding proteins with homology to *S. cerevisiae SIR2* (Fig. S2). Among these proteins, *C. albicans* Hst1 (*orf19.4762*) and Sir2 (*orf19.1992*) share the highest homology with the *S. cerevisiae* Sir2 (46% and 42% identity respectively) (Fig. S2A,B). Given the high homology, it is possible that *C. albicans* Hst1, and not Sir2, is the true ortholog of *S. cerevisiae* Sir2 as it has been demonstrated for the yeast *Clavispora lusitaniae*^11^. To determine whether Hst1 (*orf19.4762*) and/or Sir2 (*orf19.1992*) are required for maintaining the repressive state of the rDNA NTS region, we constructed *hst1* Δ/Δ and *sir2* Δ/Δ null mutants in strains carrying the *rDNA::URA3*^+^ reporter and performed marker gene silencing assay. At the NTS region of the *rDNA* locus, silencing was not alleviated in *hst1* Δ/Δ cells (Fig. 2A) but it was strongly alleviated in *sir2* Δ/Δ cells (Fig. 2B). In agreement with these results, *qRT-PCR* analyses revealed that *URA3* mRNA levels in the *rDNA::URA3*^+^ strain were significantly higher in *sir2* null cells relative to wild-type (WT) cells (Fig. 2C). Thus, the histone deacetylase Sir2, but not Hst1, is critical for the maintenance of the transcriptionally silent state associated with *rDNA* repeats in *C. albicans*. In *S. cerevisiae*, heterochromatin associated with the NTS region of the rDNA locus has been shown to repress transcription of a non-coding RNA^20^,^30^,^31^. In *C. albicans*, genome-wide analyses have identified an uncharacterised non-coding RNA (Novel_Ca21ChrR_093) originating from the NTS region of the rDNA locus^32^. To test whether *C. albicans* heterochromatin represses transcription of this non-coding RNA, we isolated RNA from wild-type and *sir2* Δ/Δ cells and performed *qRT-PCR* analyses. Deletion of the *SIR2* gene results in a clear upregulation of this non-coding transcript (Fig. 2D). Therefore, Sir2 is required to repress transcription of an endogenous transcript as well as inserted marker genes at the rDNA locus. To assess whether the NTS region of the rDNA locus is associated with heterochromatic pattern of histone marks (hypoaceylation of H3K9/H4K16 and hypomethylation of H3K4), we monitored the presence of these histone modifications by quantitative Chromatin ImmunoPrecipitation (*qChIP*)^33^. The *rDNA* locus, but not the euchromatic *ACT1* locus, showed low enrichment for H3K9 acetylation, H4K16 acetylation and H3K4 methylation (Fig. 2E–G), a chromatin state typical of heterochromatic regions. Sir2 deacetylates lysine 9 on histone H3 and/or lysine 16 on histone H4^8^,^34^,^35^. Therefore, we compared the level of histone acetylation associated with the NTS rDNA region in WT and *sir2* Δ/Δ cells by performing *qChIP* analyses. In the *sir2* null strain we detected higher levels of H3K9Ac, but not of H4K16Ac (Fig. 2E,F) demonstrating that *C. albicans* Sir2 is required to maintain low levels of acetylated H3K9. This is consistent with the idea that disruption of H3K9 deacetylation is critical for maintaining the transcriptionally silent state associated with heterochromatic regions. We also compared the level of H3K4 methylation associated with the NTS region of the rDNA locus in WT and *sir2* Δ/Δ isolates. H3K4 methylation levels did not increase in *sir2* Δ/Δ isolates compared to wt cells (Fig. 2G). Therefore hypomethylation of H3K4 is maintained independently of the histone acetylation and transcriptional state of the NTS region. Taken together these observations demonstrate that heterochromatin exists in *C. albicans* and it is assembled over the NTS region of the *rDNA* locus. Histone modification by Sir2 is critical for the maintenance of the transcriptionally silent heterochromatic state associated with this locus.

### MRS repeats are assembled into transcriptionally permissive chromatin bearing euchromatic and heterochromatic histone modifications

**Figure 3:**
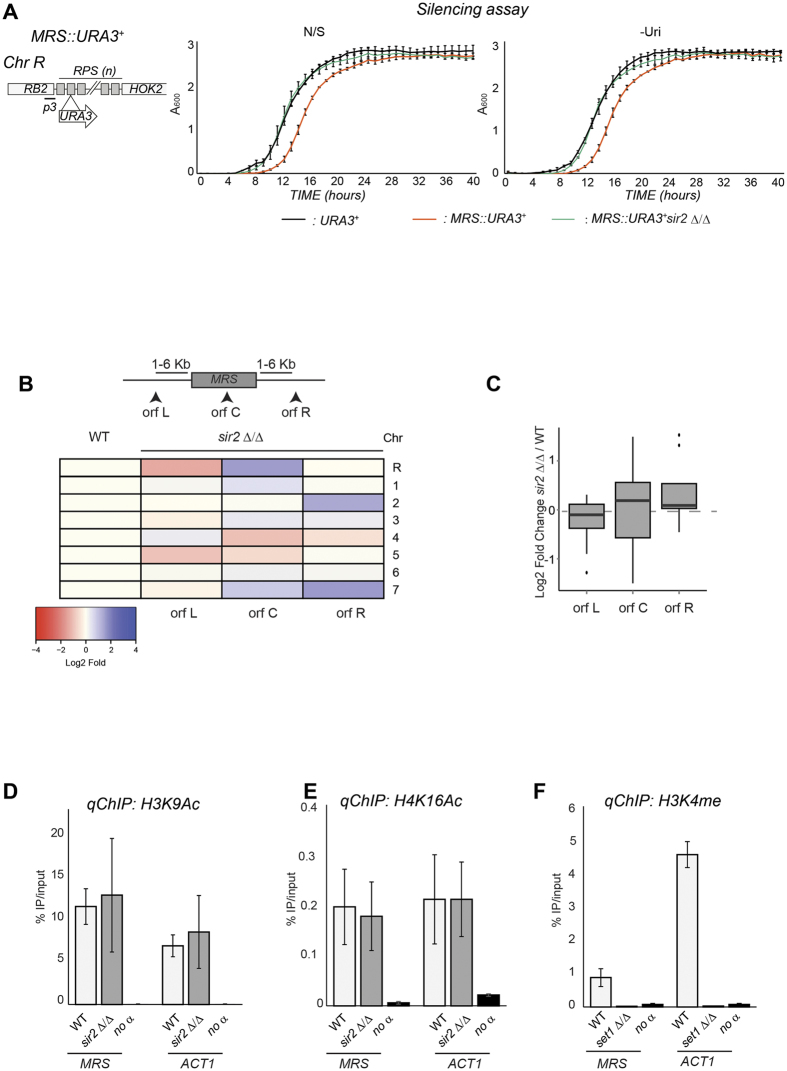
MRS repeats are assembled into transcriptionally-permissive chromatin. (**A**) *Top panel*: Schematic of *MRS::URA3*^+^ reporter strain. *Bottom panel*: Silencing assay of the *MRS::URA3*^+^ reporter strain in WT and *sir2* Δ*/*Δ isolates. A URA^+^ (*URA3*^+^) strain was included as a control. (**B,C**) RNA deep-sequencing of *sir2* Δ/Δ and WT isolates. (**B**) Normalised read counts (FPKM) of MRS associated (MRS-C) and proximal (MRS-L and MRS-R) genes were calculated from RNA-seq data for WT and *sir2* Δ/Δ isolates. The heat-map depicts the log2 fold ratio of FPKM data between *sir2* Δ/Δ and WT isolates. (**C**) Boxplot showing log2 fold changes in transcriptional expression for MRS-internal (MRS-C) and adjacent (MRS-L and MRS-R) genes between sir2 Δ/Δ and WT isolates. (**D,E**) *qChIP* to detect H3K9Ac, H4K16Ac levels associated with the *MRS* repeats and *ACT1* in WT and *sir2* Δ*/*Δ isolates and (**F**) H3K4me2 levels associated with the *MRS* repeats and *ACT1* in WT and *set1* Δ*/*Δ isolates. Error bars in each panel: Standard deviation (SD) of three biological replicates.

Having established that transcriptionally silent heterochromatin exists in *C. albicans* and is associated with the NTS region of the rDNA locus, we analysed the chromatin state associated with the MRS repeats by assessing silencing of a marker gene inserted into the tandem RPS repeats (*MRS::URA3*^+^) (Fig. 3A). As shown in Fig 3A, the *MRS::URA3*^+^ is not silenced (Fig. 3A). If the MRS repeats are associated with Sir2-dependent heterochromatin, the genes close to these regions should be upregulated in *sir2* Δ/Δ isolates compared to WT cells. To address this question, we isolated RNA from WT and *sir2* Δ/Δ cells and performed RNA-seq analyses. FPKM (fragments per kilobase of exons per million mapped reads) were determined for all the genes proximal to MRS repeats and compared in *sir2* Δ/Δ and WT strains. Upon deletion of the *SIR2* gene, we did not observe any clear effect on expression of MRS associated (orf C) and proximal genes (orf L and R) as only 3 out of 24 genes were expressed at more than 2 fold of WT in *sir2* Δ/Δ isolates (Fig. 3B and Table S5). Averaging the log2 fold change of the MRS-associated genes confirms these results (average log_2_ fold change = 0) (Fig. 3C). These data demonstrate that MRS repeats do not impose a Sir2-dependent transcriptionally repressive state.

Acetylated H3K9 and H4K16 *qChIP* analyses demonstrated that MRSs are assembled into highly acetylated chromatin where H3K9 and H4K16 are acetylated to a level similar to the active and euchromatic locus actin 1 (*ACT1*) (Fig. 3D,E). Histone acetylation level associated with these regions are similar in WT and *sir2* Δ/Δ isolates (Fig. 3D,E). In contrast, levels of H3K4 methylation associated with MRS repeats are strikingly low compared to the euchromatic *ACT1* locus (Fig. 3F). Therefore, MRS repeats are not assembled into classical transcriptionally silent heterochromatin but they are associated with an intermediate chromatin state bearing features of euchromatin (high histone acetylation) and heterochromatin (H3K4 hypomethylation).

### Heterochromatin at *C. albicans* telomeric regions

**Figure 4:**
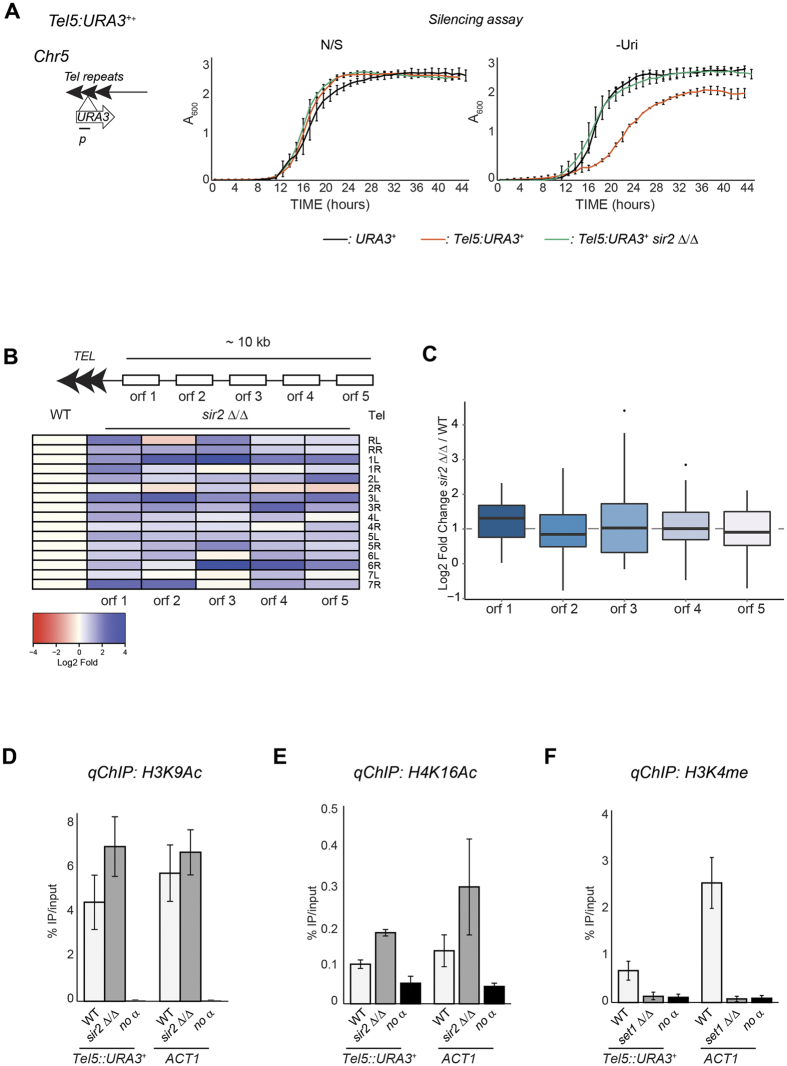
Heterochromatin at *C. albicans* telomeric regions. *Left panel*: Schematic of *Tel5::URA3*^+^ reporter strain. *Right panels*: Silencing assay of the *Tel5::URA3*^+^ reporter strain in WT and *sir2* Δ*/*Δ. (**A**) URA^+^ (*URA3*^+^) strain was included as a control. (**B,C**) RNA deep-sequencing of *sir2* Δ/Δ and WT isolates. (**B**) Normalised read counts (FPKM) of subtelomeric genes were calculated from RNA-seq data for WT and *sir2* Δ/Δ isolates. The heat-map depicts the log2 fold ratio of FPKM data between *sir2* Δ/Δ and WT isolates. (**C**) Boxplot showing log2 fold changes in transcriptional expression of subtelomeric genes between sir2 Δ/Δ and WT isolates (**D–F**) *qChIP* to detect H3K9Ac and H4K16ac levels associated with *Tel5::URA3*^+^ and *ACT1* in WT and *sir2* Δ*/*Δ strains. (**C**) H3K4me2 levels associated with *Tel5::URA3*^+^ and *ACT1* in WT and *set1* Δ*/*Δ isolates. Error bars in each panel: Standard deviation (SD) of three biological replicates.

In many organisms, heterochromatin assembly over telomeric regions is dependent not only on Sir2 but also on the Sir silencing-complex^1^,^2^. With the exception of Sir2, BLAST analyses fail to identify orthologues of telomeric silencing proteins in *C. albicans*. It is therefore possible that telomeres are not assembled into heterochromatin. To test this hypothesis, we analysed silencing of a *URA3*^+^ marker gene integrated into a telomeric region (*Tel5::URA3*^+^). The *Tel5::URA3*^+^ marker gene displayed a small but reproducible reduction in growth rate on -Uri media compared to N/S media, indicative of weak silencing (Fig. 4A). Sir2 is required to maintain this transcriptionally repressive state as silencing is alleviated in *sir2* Δ/Δ cells (Fig. 4A). Consistent with the growth assays, the levels of *URA3*^+^ mRNA levels are low in WT cells and dramatically increased in sir2 Δ/Δ isolates (Fig. S3).

Telomeric heterochromatin has been shown to repress gene expression of proximal genes in a Sir2-dependent manner^36^,^37^,^38^,^39^. To assess whether the weak marker gene silencing associated with telomere 5 is a general property of all *C. albicans* telomeres and if it extends over subtelomeric regions, we analysed the transcriptional profile of coding and non-coding subtelomeric transcripts in WT and *sir2* Δ/Δ isolates. This analysis reveals that deletion of *SIR2* results in transcriptional upregulation of many subtelomeric genes (Fig. 4B and Table S6). Although not all the subtelomeric genes are upregulated to the same extent, on average deletion of Sir2 results in a 2 fold upregulation of telomeric-proximal genes compared to WT (Fig. 4C). We used *qRT-PCR* analyses with primers specific for each subtelomeric gene to validate the difference in gene expression between WT and *sir2* Δ/Δ isolates. All the genes tested were more highly expressed in *sir2* Δ/Δ compared to WT cells (Fig. S4). Therefore, telomeric heterochromatin silences expression of genes located in proximity (~10/15 kb) of telomeres. Telomeres are composed of a 23 bp unit tandemly repeated^23^. Their repetitive nature makes the design of suitable primers for qChIP analysis particularly challenging. Thus, we assessed the telomeric chromatin state by performing qChIP analyses with primers specific for the *Tel5: URA3*^+^ marker gene. We found that telomeric chromatin is only mildly hypoacetylated on H3K9 and H4K16 and that histone acetylation levels are increased in *sir2* Δ/Δ null mutant compared to WT cells (Fig. 4D,E). In contrast, a low level of H3K4 methylation is associated with telomeric regions (Fig. 4F).

Taken together these observations demonstrate that telomeric repeats are assembled into weak heterochromatin. This chromatin state is dependent on Sir2 and is able to silence embedded marker genes as well as native proximal genes.

### Telomeric heterochromatin is plastic and remodelled upon environmental changes

**Figure 5:**
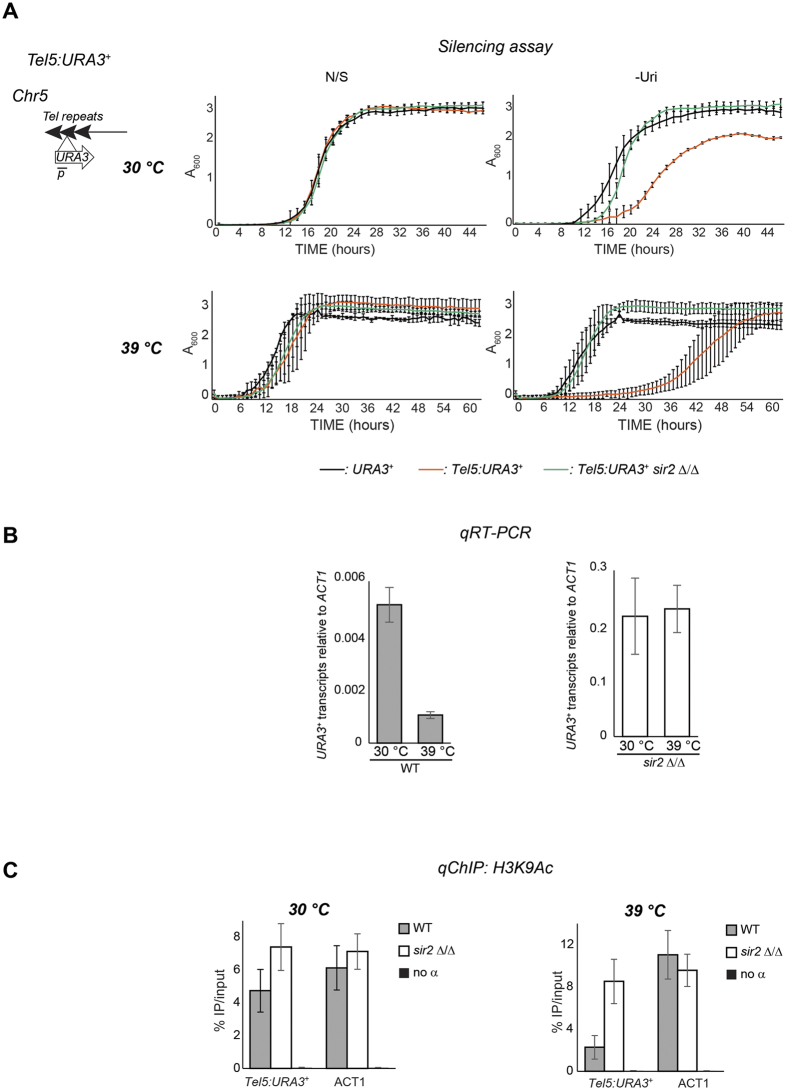
Telomeric heterochromatin is plastic. (**A**) *Left panel*: Schematic of *Tel5::URA3*^+^ reporter strain. *Right panels*: Silencing assay assessing transcriptional silencing of the *Tel5::URA3*^+^ reporter strain in WT and *sir2* Δ*/*Δ isolates at 30 °C, in the presence of 1 mM H_2_O_2_, 200 ng/μl fluconazole and at 39 °C. A URA^+^ (*URA3*^+^) strain was included as a control. (**B**) qRT-PCR analyses to measure *Tel5::URA3*^+^ transcript levels relative to *ACT1* at 30 °C and 39 °C in WT (left panel) and *sir2* Δ*/*Δ (right panel) strains. (**C**) *qChIP* to detect H3K9Ac levels associated with *Tel5::URA3*^+^ and *ACT1* in WT and *sir2* Δ*/*Δ isolats at 30 °C (Left panel) and 39 °C (Right panel). Error bars in each panel: Standard deviation (SD) of three biological replicates.

*C. albicans* is characterised by remarkable genomic and phenotypic plasticity that allow rapid adaptation to different environmental niches^4^,^16^. Therefore, we tested whether the chromatin state of *C. albicans* repetitive elements is also plastic and remodelled upon environmental changes. Remodelling could lead to dynamic chromatin structure where DNA repeats are assembled into robust transcriptionally repressive heterochromatin under specific environmental conditions and into weak heterochromatin under different environmental conditions. To test this hypothesis, we asked if physiologically-relevant stress conditions that *C. albicans* regularly encounters in the host or stress caused by antifungal agents affects the transcriptional states associated with the NTS region of the rDNA locus, telomeric repeats and MRS repeats. Silencing assays revealed that treatment with hydrogen peroxide (H_2_0_2_), mimicking the production of reactive oxygen species by the host’s immune cells, or Fluconazole, the most widely used antifungal drug, does not change the transcriptional state associated with any of the loci tested (Figs S5, S6 and S7). Likewise we found that expression of the *URA3*^+^ marker gene inserted at the rDNA locus and MRS repeats was not affected by high temperature (39 °C, mimicking moderate fever in the host) (Figs S5 and S6). In contrast, telomere-associated silencing was much stronger at 39 °C than 30 °C (Fig. 5A) indicating the telomeric chromatin is plastic. Growing cells at 39 °C did not strongly induce hyphal formation in the time frame of the experiment (Fig. S8). Therefore, the observed stronger silencing is not a consequence of a different morphology. To assess whether temperature-dependent silencing is a general feature of all *C. albicans* telomeres, we analysed silencing of a second reporter strain with a *URA3*^+^ gene integrated at the telomeric repeats of chromosome 7 left arm (*Tel7::URA3*^+^). As with the telomere repeat tract on Chr5, silencing of telomeric repeats on Chr7 was much stronger at 39 °C compared to 30 °C (Fig. S8). Importantly, silencing was dependent on Sir2 as silencing growth rate in –Uri was increased in *sir2* Δ/Δ cells (Fig. 5A). These results were confirmed by analyses of *URA3*^+^ mRNA levels by qRT-PCR: in WT, but not in *sir2* Δ/Δ cells, *Tel5: URA3*^+^ RNA levels were lower at 39 °C compared to 30 °C (Fig. 5B). Consistent with the silencing assays, *qChIP* of the *TEL5::URA3*^+^ marker revealed lower levels of H3K9 acetylation at 39 °C compared to 30 °C (Fig. 5C). Thus telomere repeats are silenced to a greater degree at febrile temperature than at 30 °C, a temperature considered relevant for growth of *C. albicans* on the skin. These results indicate that telomeric heterochromatin is dynamic and can be remodelled in a Sir2-dependent manner in response to an environmental change.

## Discussion

This study provides the first comprehensive analysis of the chromatin state of *C. albicans* DNA repeats. Our data demonstrate that, in *C. albicans*, differential chromatin states control gene expression and epigenetic plasticity is linked to adaptation to a specific environmental niche.

We find that the NTS region of the *rDNA* locus maintains a *bona fide* silent heterochromatin state that represses coding and non-coding transcription and is marked by the histone modification pattern typical of heterochromatic regions: hypoacetylation of histone H3K9 and histone H4K16 together with hypomethylation of H3K4 (Fig. 1). We identify Sir2 (*orf19.1992*) as one of the key enzymes necessary to maintain this chromatin state (Fig. 2). In *S. cerevisiae*, Sir2 targeting to the rDNA locus is dependent on the RENT complex^13^,^14^,^40^. BLAST analyses identify orthologues of the RENT complex components Net1 (*orf19.267*) and Cdc14 (*orf 19.4192*). Therefore, it is very likely that, as observed in *S. cerevisiae*, a conserved RENT complex targets heterochromatin at the *C. albicans* rDNA locus. However, the mode of action of Sir2 in *C. albicans* differs from *S. cerevisiae*: while at *S. cerevisiae* rDNA locus, Sir2 specifically deacetylates K16 on histone H4, *C. albicans* Sir2, reduces H3K9 acetylation. Therefore, heterochromatin at the non-transcribed regions of the rDNA array seems ubiquitous but it can differ in its structure.

We demonstrated that the MRS repeats are assembled into chromatin hypomethylated on H3K4, as observed in heterochromatic regions, but highly acetylated, as in euchromatic regions. Low levels of H3K4 methylation are not sufficient to create a transcriptionally repressive environment as a marker gene inserted at MRS repeats is not silenced. In addition, expression of MRS-proximal genes is not regulated by Sir2 (Fig. 3).

In many organisms, insertion of artificial DNA array is sufficient to seed heterochromatin^41^. Therefore, it is surprising that MRSs, being composed of very long tracts of nested repeats, are not assembled into classical heterochromatin. Why are MRSs not associated with heterochromatin? One primary function of heterochromatin is to inhibit recombination promoting genome stability. It is possible that hypomethylation on H3K4 is sufficient to block recombination or that an alternative mechanism promote genome stability at the MRSs.

Alternatively, genome instability at the MRSs could be beneficial for *C. albicans*, an organism lacking a canonical sexual cycle and meiosis^42^. Lack of heterochromatin at these loci could ensure high level of mitotic recombination, a key event to generate genomic diversity. In support of this hypothesis, analyses of clinical isolates suggest that MRSs might act as recombination hotspots as they can expand and contract and are known sites of translocations^21^,^43^,^44^. However it has been shown that, under standard laboratory growth conditions, recombination rate at the MRS repeats is not higher compared to a non-repetitive locus even though MRSs might have an effect on chromosome disjunction^45^,^46^ Our understanding of the biology and the function of the *MRS* loci remains limited, making it very difficult to assess whether this epigenetic signature controls *MRS* function. Further studies will reveal whether and how the epigenetic state associated with these repetitive elements contributes to *C. albicans* biology.

Traditionally, telomeric heterochromatin has been described as a repressive chromatin structure that silences expression of subtelomeric genes^2^. Recent studies have challenged this hypothesis and highlighted the diversity of structure and functions of telomeric chromatin across organisms. For example, in *S. cerevisiae* the Sir silencing complex is responsible for the assembly of hypoacetylated telomeric heterochromatin^1^. However, this chromatin state has a limited ability to repress transcription of subtelomeric genes^47^. The yeast *C. lusitaniae* appears to lack telomeric heterochromatin^11^. In the fungal pathogen *Cryptococcus neoformans* telomeric chromatin is methylated on K27 of histone H3 and silences expression of genes located in a 40 kb subtelomeric region^48^. We find that, *C. albicans* telomeres have also a specialised chromatin structure. Despite the apparent absence of Sir silencing proteins, *C. albicans* telomeres are associated with transcriptionally repressive heterochromatin (Fig. 5). Sir2 (*orf19.1992*) controls heterochromatin assembly at telomeres by deacetylation of histones. We hypothesise that an as yet unidentified protein complex targets Sir2 to telomeres. We demonstrate that telomeric heterochromatin transcriptionally silences subtelomeric genes as deletion of the *SIR2* gene causes their upregulation. This modulation is likely to have major impacts on the biology and the pathogenicity of *C. albicans* as many subtelomeric genes have key regulatory functions. For example, the subtelomeric *TLO* genes encode proteins with similarity to Med2, a component of the Mediator complex that regulates transcription by RNA polymerase II^49^ and the subtelomeric gene Nag4 encodes for a putative transporter^50^.

We find that telomeric heterochromatin is dynamic: the ability of telomere terminal repeats to repress the expression of an embedded *URA3* gene is affected by a physiologically-relevant stress condition with silencing much stronger at a temperature (39 °C) mimicking fever in the host than at lower temperatures (30 °C). This enhanced silencing is linked to changes in chromatin structure, as telomeres at 39 °C had lower levels of H3K9 acetylation and Sir2 was required for the increased silencing. This effect is specific for telomeric regions and for temperature shift as other loci are not affected by temperature changes and other physiologically relevant stresses do not lead to chromatin remodelling. Therefore, in *C. albicans*, adaptation to higher temperature is linked to chromatin remodelling at telomeric regions.

Dynamic heterochromatin is seen at telomeres in many species: For example, in *S. cerevisiae*, transcriptional silencing at higher temperature is enhanced at telomeres and weaker at the *rDNA* locus^51^. This could be due to a temperature-sensitive protein important for telomere function^52^ or to changes in the levels of heat shock factors at different temperatures^53^. The dynamics of heterochromatin also affects virulence and pathogenesis in at least some microbial pathogens. For example, telomeric heterochromatin regulates the expression of the antigenic variation gene in parasites^38^. The biological consequences of telomere chromatin plasticity in *C. albicans* remain to be determined. It is possible that changes in telomeric heterochromatin correlate with changes in expression of subtelomeric genes. Alternatively, chromatin remodelling at telomeres could regulate genomic stability of these loci.

In conclusion, this study highlights the diversity and plasticity of chromatin states associated with DNA repeats in *Candida albicans*, the most common human fungal pathogen. We show that in *C. albicans* differential heterochromatin states control gene expression independently of the underlying DNA sequence and remodelling of heterochromatin is linked to adaptation in a stress condition.

## Methods

### Growth conditions

Yeast cells were cultured in rich medium (YPAD) containing extra adenine (0.1 mg/ml) and extra uridine (0.08 mg/ml), complete SC medium (Formedium^TM^) or SC Drop-Out media (Formedium^TM^). Cells were grown at 30 °C or 39 °C as indicated.

### Yeast strain construction

Strains are listed in Supplementary Table S1. Integration and deletion of genes were performed as previously described^54^. Oligonucleotides and plasmids used for strain constructions are listed in Supplementary Table S2 and Supplementary Table S4, respectively. Transformation was performed by electroporation (Gene Pulser^TM^, Bio-Rad) using the protocol described in^55^. Correct integration events were checked by PCR and/or Southern blotting using primers listed in Supplementary Table S2 (Fig. S9)

### Silencing assay

Growth analyses with *rDNA::URA3*^+^, *Tel5::URA3*^+^ and *MRS::URA3*^+^ strains were performed using a plate reader (SpectrostarNano, BMG labtech) in 24 well or 96 well plate format at 30 °C. When indicated Silencing assays were performed in the presence of 200 ng/μl of fluconazole (Sigma), 1 mM H_2_0_2_ (Sigma) and 39 °C. For each silencing assay in a 24 well plate format, 1 ml of a starting culture was inoculated in SC or SC-Uri media to reach a concentration of 60 cells/μl. Growth was assessed by measuring A_600_, using the following conditions: OD600 nm, 3600 s cycle time, 30 flashes per well, 400 rpm shaking frequency, double orbital shaking mode, 850 s additional shaking time after each cycle, 0.5 s post delay, for 44 to 60 hours at 30 °C. For each silencing assay in 96 well plate format, 1:100 dilution of an starting culture was inoculated in a final volume of 95 μl of SC or SC-Uri media to reach a concentration of 60 cells/μl. Growth was assessed by measuring A_600_, using the following conditions: OD600 nm, 616 cycle time, 3 flashes per well, 700 rpm shaking frequency, orbital shaking mode, 545 s additional shaking time after each cycle 0.5 s post delay, for 44 hours.

Graphs represent data from three biological replicates. Error bars: standard deviations of three biological replicates. Data was processed using SpectrostarNano MARS software and Microsoft Excel.

### RNA extraction and cDNA synthesis

All strains were grown in YPAD rich media. RNA extraction was performed using a yeast RNA extraction kit (E.Z.N.A.® Isolation Kit RNA Yeast, Omega Bio-Tek) following the manufacturer’s instructions. RNA quality was checked by electrophoresis under denaturing conditions in 1% agarose, 1× HEPES, 6% Formaldehyde (Sigma). RNA concentration was measured using a NanoDrop ND-1000 Spectrophotometer. cDNA synthesis was performed using iScript™ Reverse Transcription Supermix for RT-qPCR (Bio-Rad) following manufacturer’s instructions and a Bio-Rad CFXConnect^TM^ Real-Time System.

### High-throughput RNA sequencing

Strand-specific cDNA Illumina Barcoded Libraries were generated from 1 μg of total RNA extracted from WT and *sir2* Δ/Δ and sequenced with an Illumina iSeq2000 platform. Illumina Library and Deep-sequencing was performed by the Genomics Core Facility at EMBL (Heidelberg, Germany). Raw reads were analysed following the RNA deep sequencing analysis pipeline described^56^ using Galaxy (https://usegalaxy.org/) and Linux platform. Heatmaps and boxplot graphs were generated with R (http://www.r-project.org/). RNA sequencing data are deposited into ArrayExpress (accession number E-MTAB-4488).

### Quantitative Chromatin ImmunoPrecipitation (qChIP)

qChIP was performed as described^33^ with the following modifications: 5 ml of an overnight culture grown in YPAD with extra uridine (0.08 mg/ml), diluted into fresh YPAD with extra uridine (0.08 mg/ml) and grown until OD600 nm of 1.4. Cells (50 ml/sample) were fixed with 1% Paraformaldehyde (Sigma) for 15 min at room temperature. Cells were lysed using acid-washed glass beads (Sigma) and a Disruptor genie^TM^ (Scientific Industries) for 30 min at 4 °C. Chromatin was sheared to 500–1000 bp using a Bioruptor (Diagenode) for a total of 20 min (30 s ON and OFF cycle) at 4 °C. Immunoprecipitation was performed overnight at 4 °C using 2 μL of antibody anti-H3K4me2 (Active Motif- Cat Number: 39141), anti-H3K9ac (Active Motif- Cat Number: 39137), and anti-H4K16ac (Active Motif- Cat Number: 39167) and 25 μl of Protein G magnetic beads (Dynal - InVitrogen). DNA was eluted with a 10% slurry of Chelex 100-resin (Bio-Rad) using the manufacturer’s instructions.

### qPCR reactions

Primers used are listed in Supplementary Table S3. Real-time qPCR and RT-qPCR was performed in the presence of SYBR Green (Bio-Rad) on a Bio-Rad CFXConnect^TM^ Real-Time System. Data were analysed with Bio-Rad CFX Manager 3.1 software and Microsoft Excel. Enrichments were calculated as the percentage ratio of specific IP over input for qChIP analysis and as enrichment over actin for RT-qPCR. Histograms represent data from three biological replicates. Error bars: standard deviation of three biological replicates.

### Southern blot

Genomic DNA was extracted using glass acid beads (Sigma), phenol: cholorform: isoamyl alcohol (25:24:1) (Sigma) and RNAse A treated (Fisher). Following centrifugation, pellets were precipitated with 0.05 mM Sodium Acetate (Sigma) and Ethanol (Fisher) at −20 °C during 30 minutes and resuspended in water.Genomic DNA was then digested with corresponding enzymes and run in 1% agarose gel. DNA was transferred to a nylon membrane (Zeta probe membranes, Bio-Rad), probed with DIG probes (Roche) and hybridized as described^57^.

### Microscopy

Microscopy was carried out using an Olympus IX81 inverted microscope. Images were captured with a Hamamatsu photonics C4742 digital camera, with light excitation from an Olympus MT20 illumination system. Olympus CellR imaging software was used to control the apparatus.

## Additional Information

**How to cite this article**: Freire-Benéitez, V. *et al.* *Candida albicans* repetitive elements display epigenetic diversity and plasticity. *Sci. Rep.* **6**, 22989; doi: 10.1038/srep22989 (2016).

## Supplementary Material

Supplementary Information
