# Supplementary material for: Candida albicans repetitive elements display epigenetic diversity and plasticity
Source: Sci Rep. 2016 Mar 14;6:22989. doi: 10.1038/srep22989 (PMC4789652; doi:10.1038/srep22989)
Supplement: Supplementary Information [file srep22989-s1.pdf]

Supplementary Information

Candida albicans repetitive elements display epigenetic diversity and plasticity

Verónica Freire-Benéitez, Robert Jordan Price, Daniel Tarrant, Judith Berman and Alessia Buscaino

Content:

Supplementary Figures S1 to S9 with Legends

Supplementary Tables S1 to S6

Supplementary Figures

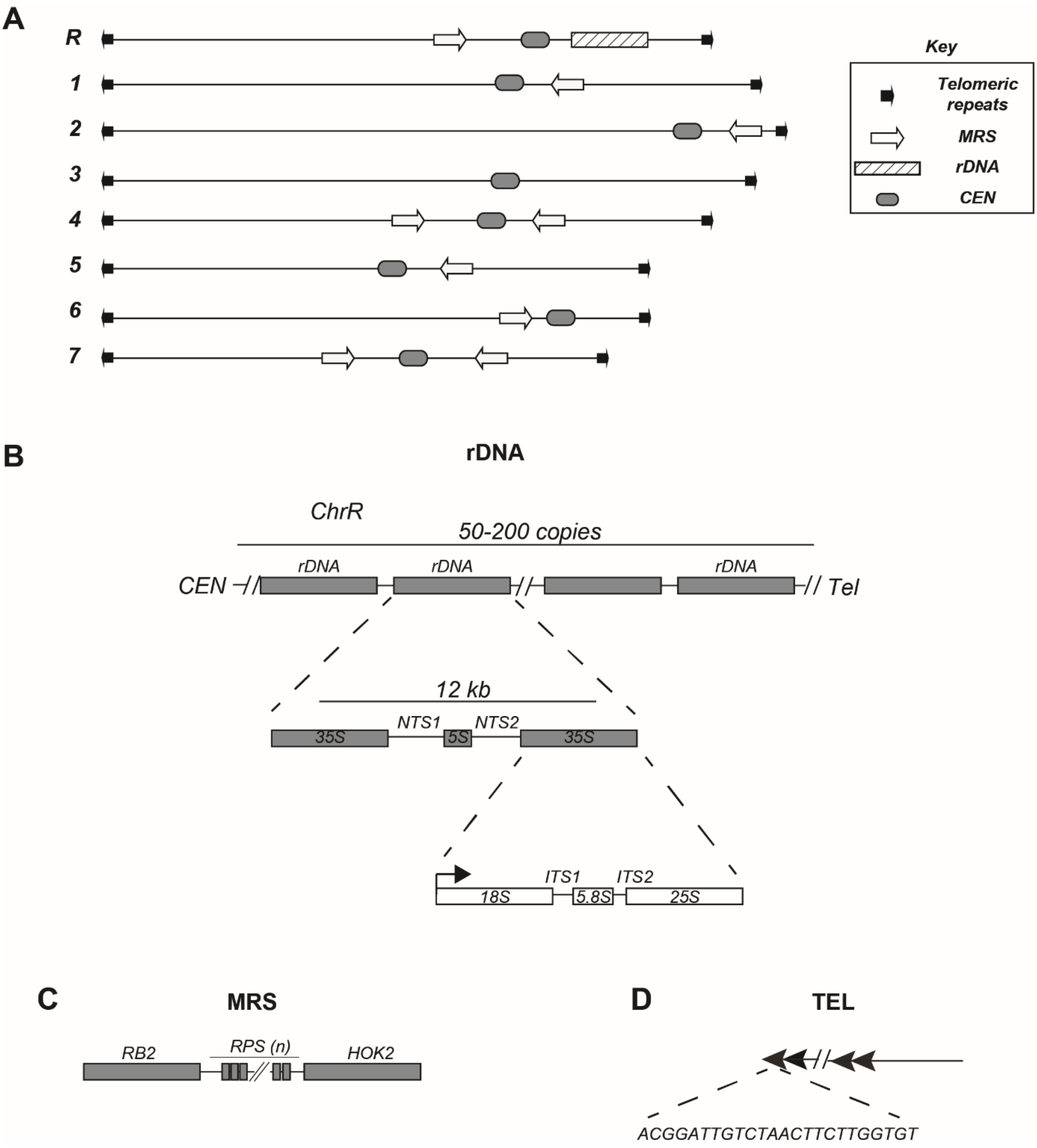

Supplementary Figure 1 C. albicans DNA repeats.

(A) Schematic of *Candida albicans* repeats. The cluster of ribosomal DNA repeated genes (rDNA) is located on chromosome R. Major Repeated Sequences (MRS) are located in all chromosomes except chromosome 3. Telomeric repeats are present at the end of each chromosome. **(B)** Organisation of the rDNA locus in *C. albicans*. The rDNA locus is formed by tandem arrays of a 12 kb unit repeated 50 to 200 times on chromosome R. Each unit

contains the 35 S and the 5S rRNA genes that are separated by two Non-Transcribed Regions (NTS1 and NTS2). The 35 S gene is the precursor of the 18S, 5.8S and 28S rRNAs that are separated by ITS (Internal Transcribed Spacer) elements **(C)** Organisation of MRS repeats. The MRS repeats are formed by tandem arrays of a 2.1 kb unit (RPS) flanked by HOK and RBP-2 elements. **(D)** Organisation of Telomeric repeats. Telomeric repeats are found at the end of each chromosome and are composed of tandemly repeating 23 bp unit. The sequence of the unit is indicated.

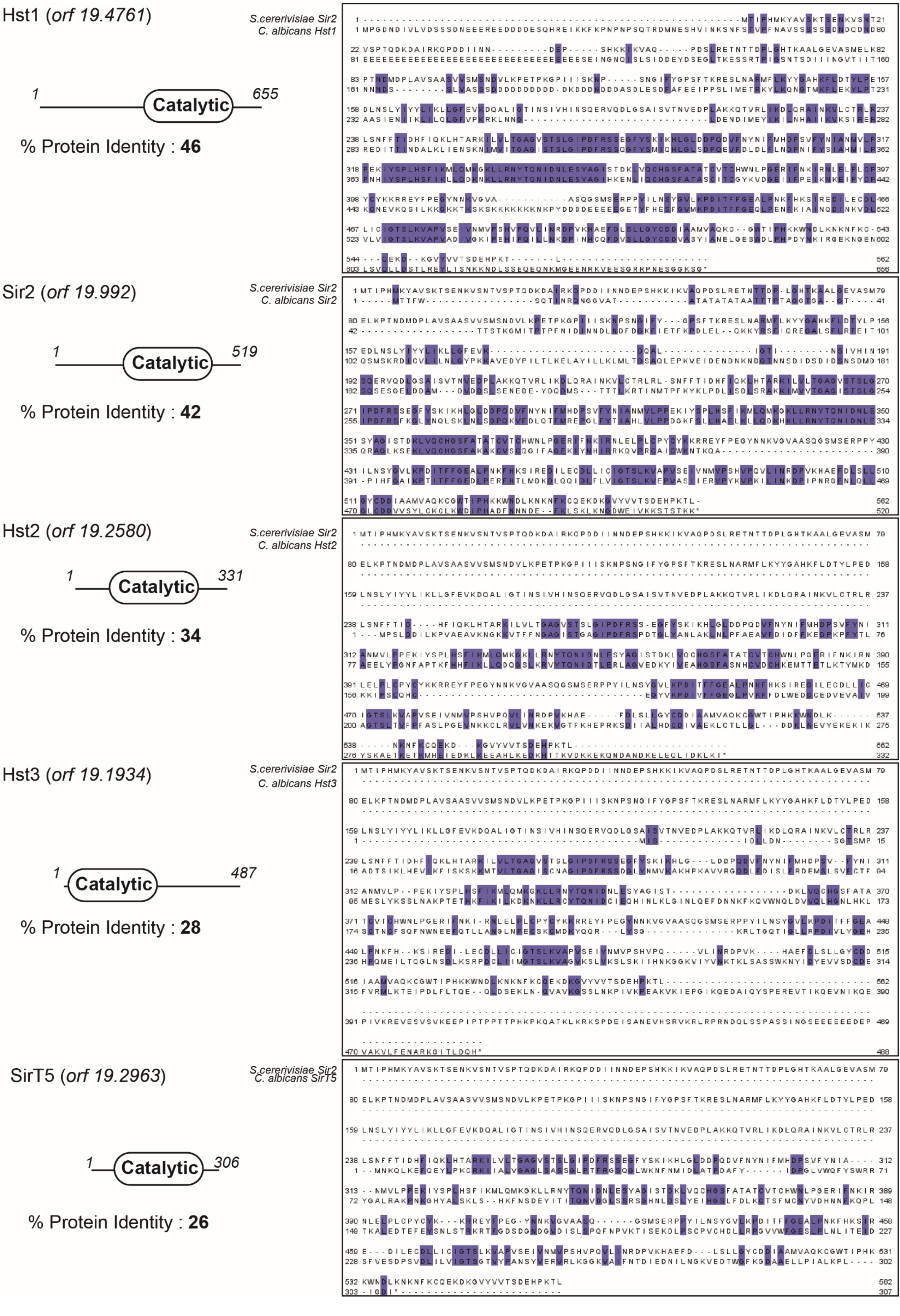

Hst1 (orf 19.4761)
1
Catalytic
655
% Protein Identity : 46
S.cererivisiae Sir2
C. albicans Hst1
Sir2 (orf 19.992)
1
Catalytic
519
% Protein Identity : 42
S.cererivisiae Sir2
C. albicans Sir2
Hst2 (orf 19.2580)
1
Catalytic
331
% Protein Identity : 34
S.cererivisiae Sir2
C. albicans Hst2
Hst3 (orf 19.1934)
1
Catalytic
487
% Protein Identity : 28
S.cererivisiae Sir2
C. albicans Hst3
SirT5 (orf 19.2963)
1
Catalytic
306
% Protein Identity : 26
S.cererivisiae Sir2
C. albicans SirT5

Supplementary Figure 2. Sir2-like proteins in *Candida albicans*

(A-E) *Left panels*: Diagram depicting protein length and domain organisation of the *C. albicans* Sir2-like proteins. The percentage (%) protein sequence identity between the *C. albicans* proteins and *S. cerevisiae* Sir2 is indicated. *Right panels*: Pairwise sequence alignments between *S. cerevisiae* Sir2 and *C. albicans* proteins with similarity to *S. cerevisiae* Sir2.

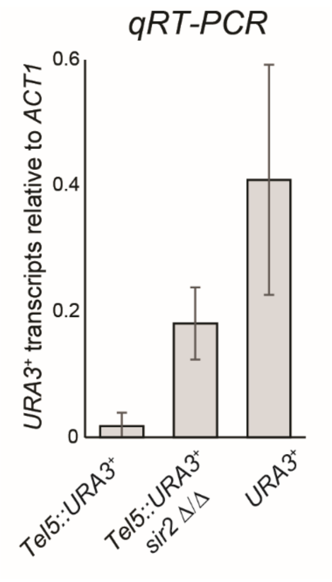

Supplementary figure 3. Transcriptional Silencing at C. albicans telomeric repeats.

qRT-PCR analyses to measure *URA3* transcript levels relative to actin transcript levels (*ACT1*) at 30°C in *Tel5::URA3* wild-type and *sir2 Δ/Δ* strains compared to *URA3* endogenous transcription levels. Error bars: SD of three biological replicates.

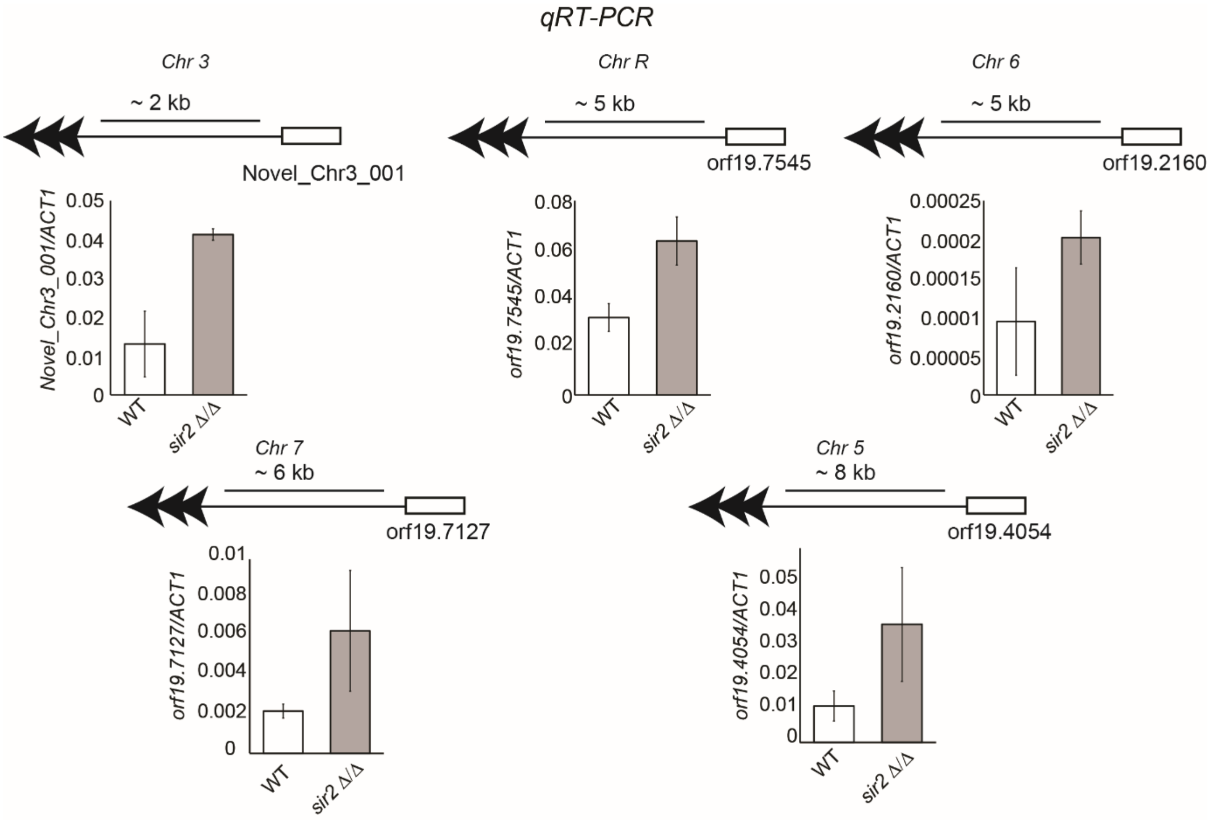

Supplementary Fig 4. qRT-PCR analyses to measure transcript levels of subtelomeric coding and non-coding transcripts (*Novel_Chr3_001, orf19.7545, orf19.2160, orf19.7127* and *orf19.4054*) relative to actin transcript levels (*ACT1*) in *Tel5::URA3* wild-type and *sir2 Δ/Δ* strains compared to *URA3* endogenous transcription levels. Error bars in each panel: Standard deviation (SD) of three biological replicates.

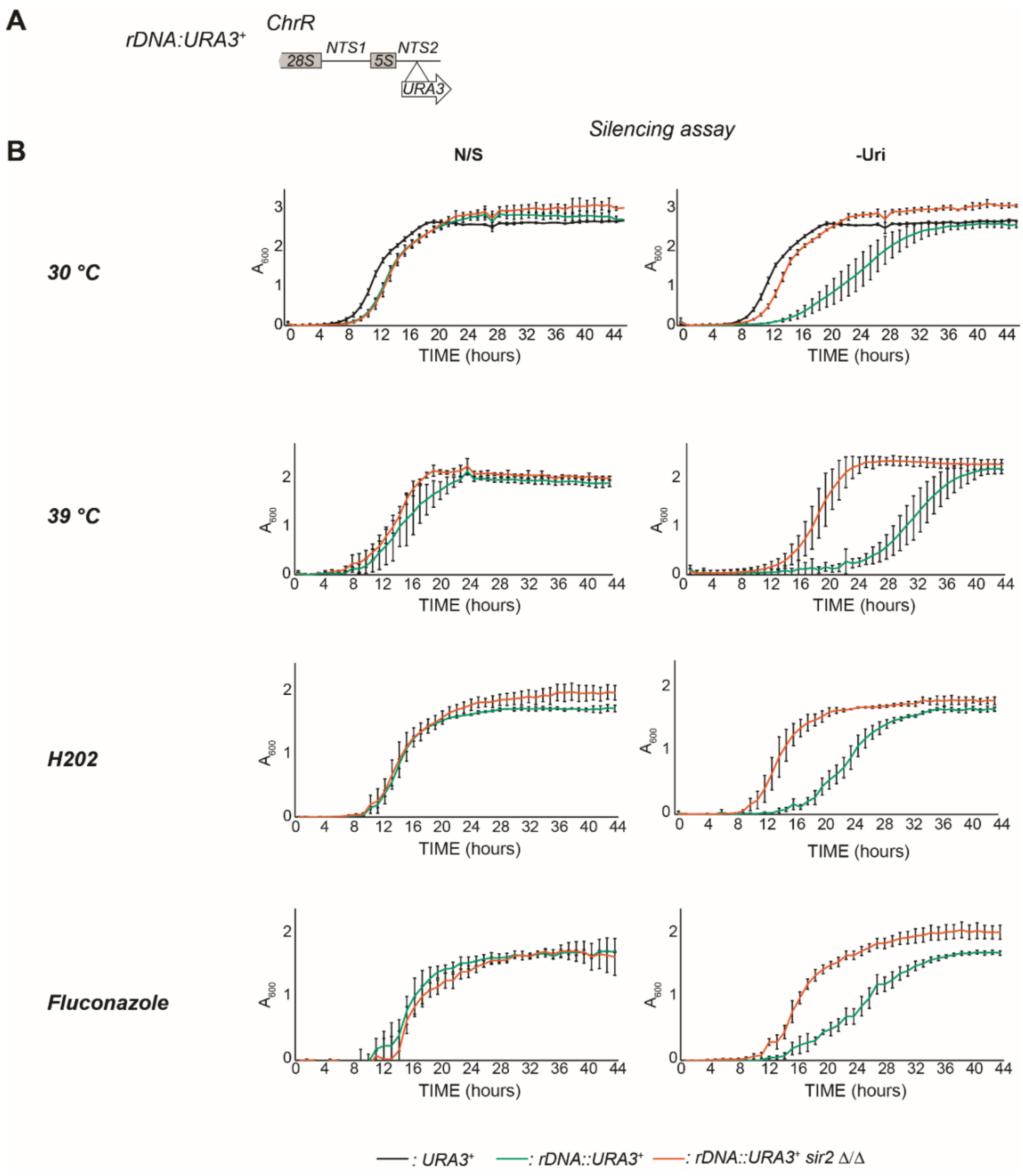

Supplementary figure 5. Environmental changes do not affect the transcriptional state of the rDNA locus

(A) Schematic of *rDNA::URA3* reporter strain. **(B)** Silencing assay assessing transcriptional silencing of the *rDNA::URA3* reporter strain in wild-type and sir2 deletion mutant (*sir2 Δ/Δ*) at 30°C, 39°C, in the presence of 1 mM H_2O_2, and 200 ng/µl fluconazole. Cells were grown in non-selective (N/S) and media lacking uridine (-Uri) and A_{600} was measured every hour for 44 hours. A URA^+ (*URA3)* strain was included as a control. Error bars: SD of three biological replicates.

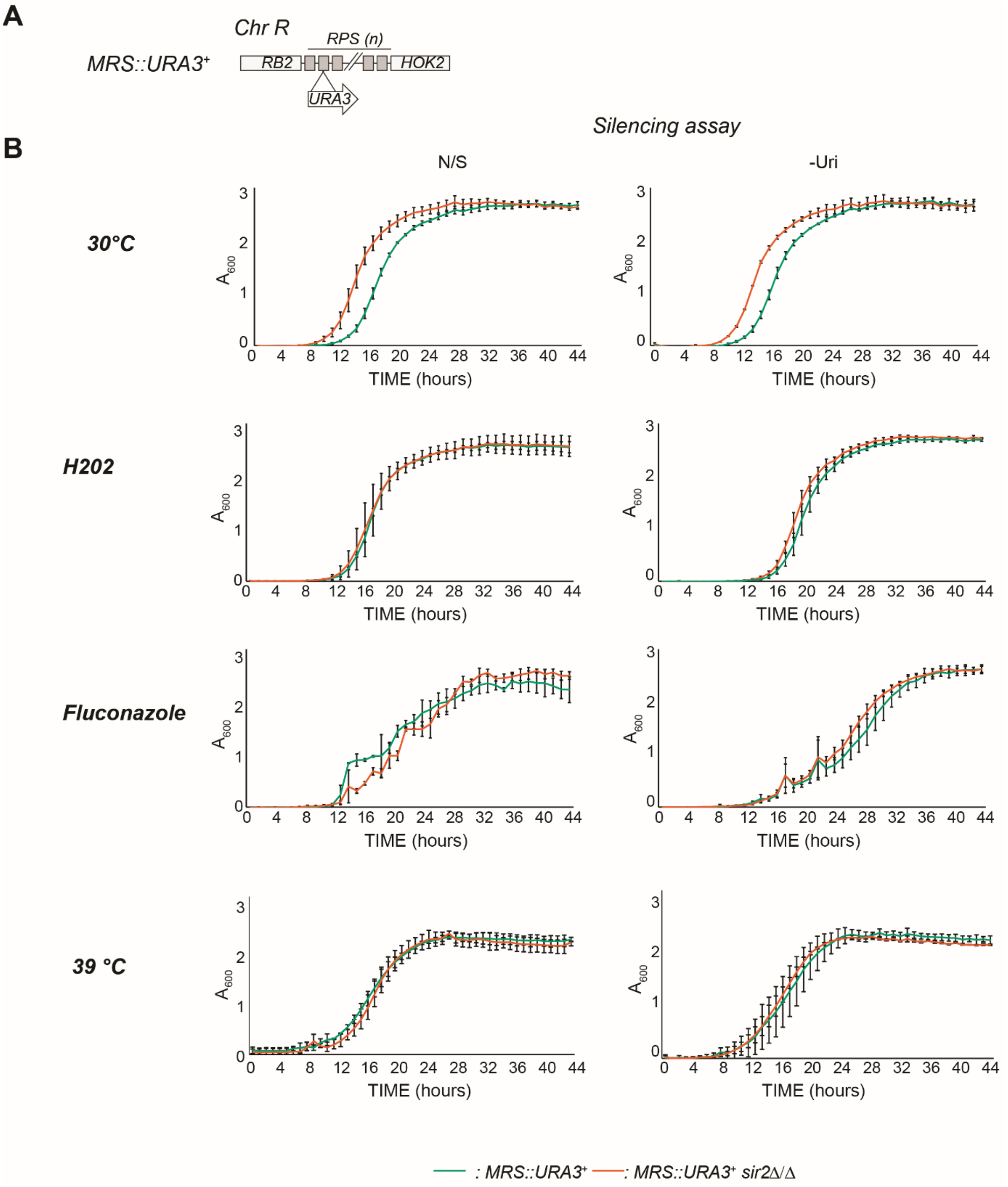

Supplementary figure 6. Environmental changes do not affect the transcriptional state of MRS repeats

(A) Schematic of *MRS::URA3*+ reporter strain. **(B)** Silencing assay assessing transcriptional silencing of the *MRS::URA3*+ reporter strain in WT and sir2 deletion mutant (*sir2 Δ/Δ*) at 30°C, in the presence of 1 mM H_2O_2, and 200 ng/µl fluconazole and at 39°C. Cells were grown in non-selective (N/S) and media lacking uridine (-Uri) and A_{600} was measured every hour for 44 hours. Error bars: SD of three biological replicates.

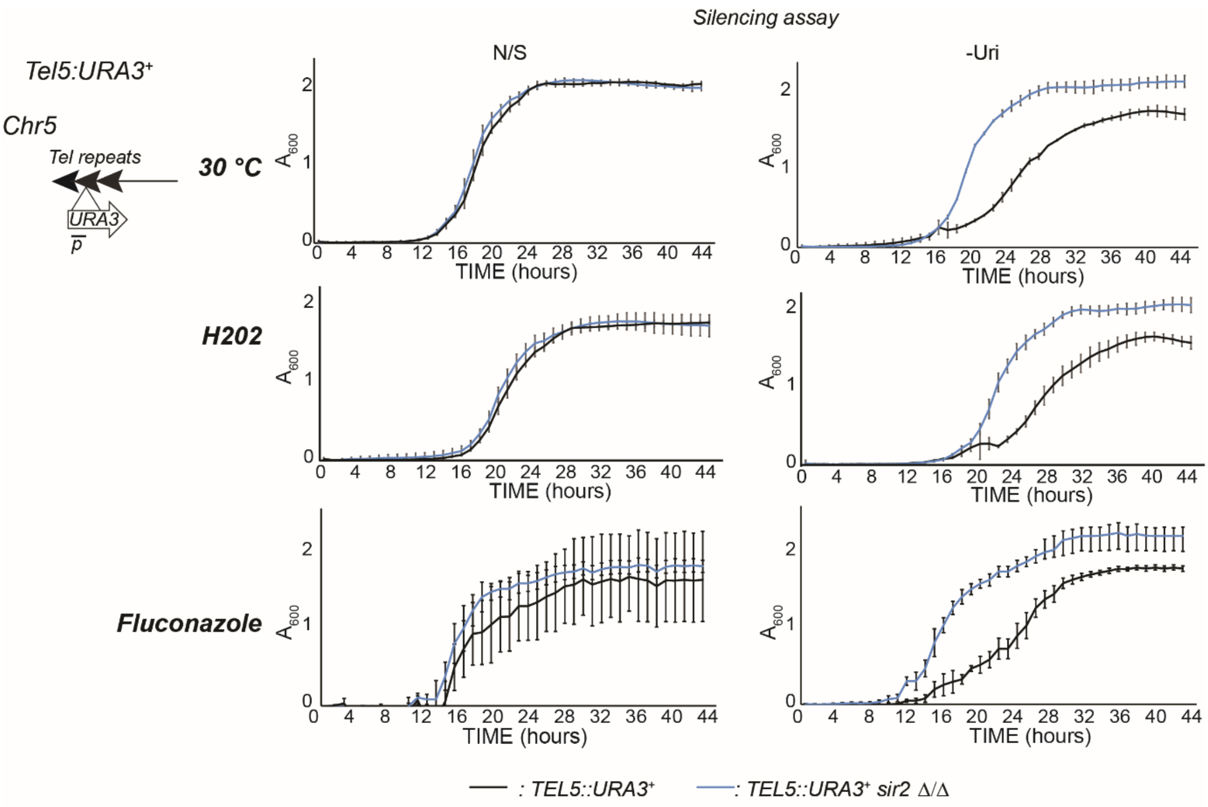

Supplementary Figure 7. H202 treatment and Fluconazole treatment does not affect silencing at telomeric regions

A) *Left panel*: Schematic of *Tel5::URA3*$^{+}$ reporter strain. *Right panels*: Silencing assay assessing transcriptional silencing of the *Tel5::URA3*$^{+}$ reporter strain in WT and sir2 deletion mutant (*sir2 Δ/Δ*) at 30°C, in the presence of 1 mM H_2O_2, 200 ng/µl fluconazole. Cells were grown in non-selective (N/S) and media lacking uridine (-Uri) and A_{600} was measured every hour for 44 to 60 hours.

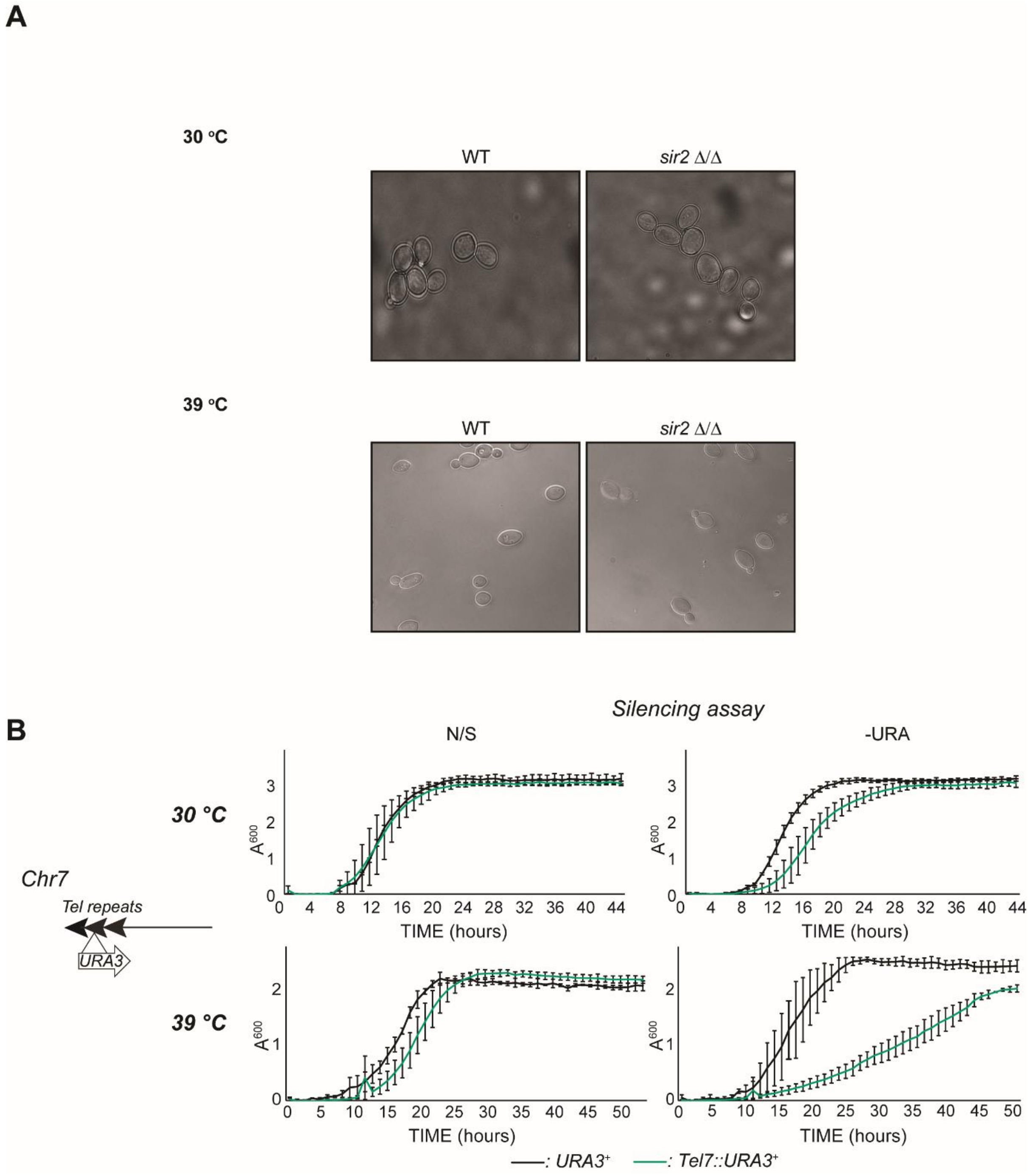

Supplementary figure 8. Plastic Heterochromatin at telomeric region

A) Cell morphology of WT and sir2 D/D strains grown at 30 °C 39 °C for 24 hours in YPAD medium. **B)** *Left panel*: Schematic of *Tel7::URA3*$^{+}$ reporter strain. *Right panels*: Silencing assay assessing transcriptional silencing of the *Tel7::URA3*$^{+}$ reporter strain at 30°C and at 39°C. Cells were grown in non-selective (N/S) and media lacking uridine (-Uri) and A_{600} was measured every hour for 50 hours. Error bars: SD of three biological replicates.

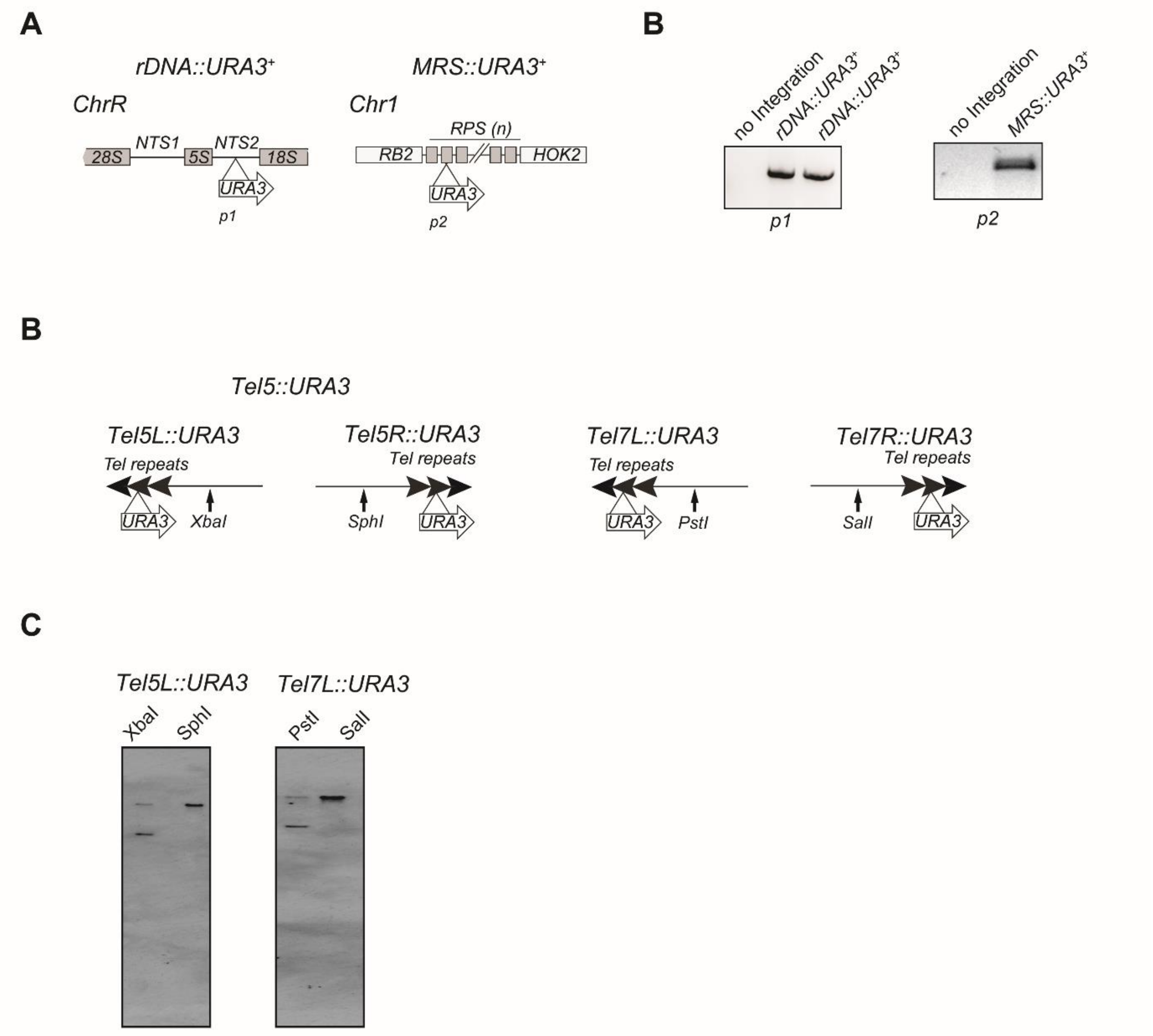

Supplementary figure 9. URA3+ marker gene reporter strains.

(A) Schematics of *rDNA::URA3*$^+$ and *MRS::URA3*$^+$. **(B)** PCR analyses confirming integration of the URA3$^+$ marker gene at the rDNA locus (*rDNA::URA3*$^+$) and the MRS repeats (*MRS::URA3*$^+$). (C) Southern blot of *C. albicans* genomic DNA digested with arm specific enzymes (*Tel5L*: *XbaI*, *Tel5R*: *SphI*, *Tel7L*: *PstI*, *Tel7R*: *SalI*) demonstrating that the URA3$^+$ gene is inserted on Chromosome 5 left arm (*Tel5L::URA3*) and Chromosome 7 left arm (*Tel7L::URA3*) . The blot was probed with a DIG probe targeting the URA3$^+$ gene.

Supplementary Table

Supplementary Table 1: Strains used in this study

Strain number	Description	Genotype
Bu_20	*sir2Δ/Δ*	*ura3Δ::λimm434/ura3Δimm434 his1::hisG/his1::hisG arg4::hisG/arg4::hisG sir2 Δ::HIS1/sir2 Δ::ARG4*
Bu_44	*Tel5:URA3*	Tel5*::URA3 ura3Δ::λimm434/ura3Δ::λimm434 his1::hisG/his1::hisG arg4::hisG/arg4::hisG*
Bu_45	*Tel5:URA3 sir2Δ/Δ*	Tel5*::URA3 ura3Δ::λimm434/ura3Δ::λimm434 his1::hisG/his1::hisG arg4::hisG/arg4::hisG sir2Δ::HIS1/sir2Δ::ARG4*
Bu_60	BWP17	*ura3Δ::λimm434/ura3Δimm434 HIS1::his1::hisG/his1::hisG ARG4::arg4::hisG/arg4::hisG*
Bu_70	*set1Δ/Δ*	*ura3Δ::λimm434/ura3Δimm434 his1::hisG/his1::hisG arg4::hisG/arg4::hisG set1 Δ::HIS1/set1 Δ::LEU2*
Bu_83	*Tel7:URA3*	Tel7*::URA3 ura3Δ::λimm434/ura3Δ::λimm434 his1::hisG/his1::hisG arg4::hisG/arg4::hisG*
Bu_95	*rDNA:URA3*	rDNA::*URA3 ura3Δ::λimm434/ura3Δimm434 his1::hisG/his1::hisG arg4::hisG/arg4::hisG*
Bu_102	*rDNA:URA3 sir2Δ/Δ*	rDNA::*URA3 ura3Δ::λimm434/ura3Δimm434 his1::hisG/his1::hisG arg4::hisG/arg4::hisG sir2Δ::HIS1/sir2Δ::ARG4*
Bu_106	*rDNA:URA3 hst1Δ/Δ*	rDNA::*URA3 ura3Δ::λimm434/ura3Δimm434 his1::hisG/his1::hisG arg4::hisG/arg4::hisG hst1Δ::HIS1/hst1Δ::ARG4*
Bu_215	BWP17	*ura3Δ::λimm434/ura3Δ::λimm434 his1::hisG/his1::hisG arg4::hisG/arg4::hisG*
Bu_227	*MRS:URA3*	MRS::*URA3 ura3Δ::λimm434/ura3Δimm434 HIS1::his1::hisG/his1::hisG ARG4::arg4::hisG/arg4::hisG*
Bu_244	*MRS:URA3 sir2Δ/Δ*	MRS::*URA3 ura3Δ::λimm434/ura3Δimm434 his1::hisG/his1::hisG arg4::hisG/arg4::hisG sir2Δ::HIS1/sir2Δ::NAT*

Supplementary table 2. Primers used in this study

Primer	Sequence	Figure	Description
Bu_91	TTACATCTTATGGCTATGAATTGGCACTAGTTGGGTCCTTCTAGCATCGAAACTGACTGTGTCAGCCTTAGCGGAGTTAATTTCACATTGTTTTCCCAGTCACGACGTT	Fig 3A, S6B	MRS::*URA3*
Bu_92	TTGAAGCTACAATTATGTAGAGTATTGGGTGTGAATTAGGCATGAATCGGATCAAAATTGGTTGAGCTATTGAAGAAAACGTTTTCTCCGTGGATGTGGAATTGTGAGCG	Fig 3A, S6B	MRS::*URA3*
Bu_98	CCTTAACTCCGTCTCCGTGT	Fig 3A, S6B	Primer to check MRS::*URA3*
Bu_121	GGAGTACTCCTATACTAATAACAAACACTCCACTATAATTGGCAACCACAATTATGCCTGGGGAAATATTGTTTTCCCAGTCACGACGTT	Fig 1A-B, 2A-C S5B	*rDNA*:*URA3*
Bu_122	AATGCACGTGACCCACACAATTTTCAACCACCAACAACACACCAAAAATGTATGTACACTCGGAGTGGAATGTGGAATTGTGAGCGGATA	Fig 1A-B, 2A-C, S5B	*rDNA*:*URA3*
Bu_131	GACTGGCCAATTATAAATGTGAAGG	Fig 1A-B, 2A-C, S5B	Primer to check rDNA:*URA3*
Bu_132	GTCTAAATTCCCCTTCCCCATAC	Fig 1A-B, 2A-C, S5B	Primer to check *rDNA*:*URA3*
Bu_135	CTAGAAATCACTAGTGCGGCC	Fig 3A, S6B	Primer to check MRS:*URA3*
Bu_139	GAGTGAGTGAGTGGAGTAGCG	Fig2B-C, Fig3A, FigS5B, FigS6B	Primer to check *sir2Δ/Δ* deletion
Bu_152	CTGGAGAAAATATAACCACGAGTCTAAGTTTCTTTATTATATTGACGTTTCAGTTATTTGAGAGAAATCCTCTAGTAGTTTTCCCAGTCACGACGTT	Fig2B-C, Fig3A, FigS5B, FigS6B	*sir2Δ/Δ* deletion mutant
Bu_153	ATATATAAATATATAAATATATATATATAAAAGAATTGAAAAGAAAAACATTAAAGACACCAATATTAATTTAATGTGGAATTGTGAGCGGATA	Fig2B-C, Fig3A, FigS5B, FigS6B	*sir2Δ/Δ* deletion mutant
Bu-158	CTATCAAAACACTCACTTAGTTACATATATATTCTTATTCTTATCAATTATTACTAATAACAAATAACAATCAATAGTTTTCCCAGTCACGACGTT	Fig2A	*hst1Δ/Δ* deletion mutant
Bu-159	ACGTCTATAGTTTATCTATCGGGGCTTTCTCTTCCTCT	Fig2A	*hst1Δ/Δ* deletion mutant

	TTGTCCTCGTTGTCCACTT TATCTTGTTTTGGCTCTTG TGGAATTGTGAGCGGATA		
Bu_164	CGGTCTGGTAAATGATTGA C	Fig2B-C, Fig3A, FigS5B, FigS6B	Primer to check *HIS1* integration
Bu_165	AGTGTGGAAAGAAGAGAT GC	Fig2B-C, FigS5B	Primer to check *ARG4* integration
Pf_169	CACCACCACTTCTACCACT TC	Fig2A	Primer to check *hst1Δ/Δ* deletion
Bu_179	CTGTATCTATAAGCAGTAT CATCC	Fig 3A, FigS5B	Primer to check *NAT* integration
Bu_286	CTGGAGAAAATATAACCAC GAGTCTAAGTTTCTTTATT ATATTGACGTTTCAGTTAT TTGAGAGAAATCCTCTAGT AGTAAAACGACGGCCAGT GAATTC	Fig 3A, FigS5B	*sir2Δ/Δ* deletion mutant
Bu_287	ATATATAAATATATAAATAT ATATATATAAAAGAATTGA AAAGAAAAACATTAAAGAC ACCAATATTAATTTAATGC ATCAATTGACGTTGATACC AC	Fig 3A, FigS5B	*sir2Δ/Δ* deletion mutant

Supplementary table 3. qPCR primer used in this study

**Primer**	**Sequence**	**Figure**	**Description**
Bu_108	GGCACTAGTTGGGTCCTT CT	Fig3D-F	MRS: qChip
Bu_109	GGGCCGTTTTGAAGCTAC AA	Fig3D-F	MRS: qChip
Bu_129	GTTGTCTGACCATGGGTA TACCA	Fig2E-G	rDNA: qChip
Bu_138	CCAGGCATAATTGTGGTT GCC	Fig2E-G	rDNA :qChip
Bu_141	GTTGGGCAGATATTACCA ATG	Fig1B,2C, 5B-C, FigS3, FigS9	*URA3* probe, *Tel5-URA3* qChip, RT-qPCR
Bu-142	CCTTCACATTTATAATTGG CC	FigS9	*URA3* probe
Bu_174	CTACGTTTCCATTCAAGCT GTT	Fig1B, Fig2C-G, Fig3D-F, Fig4D-F, Fig5B-C FigS3, FigS4	*Act1*: qChip, RT-qPCR
Bu_176	AAACTGTAACCACGTTCA GACA	Fig1B, Fig2C-G, Fig3D-F, Fig4D-F, Fig5B-C FigS3, FigS4	*Act1:* qChip, RT-qPCR
Bu_204	CAAATTCCTTATCGGATTT AGC	Fig1B,2C, 5B-C, FigS3FigS3	*URA3*, *Tel5-URA3* qChip, RT-qPCR
Bu_430	GGCAGAGGAAGCGAAGA AG	FigS4	orf19.7127 RT-qPCR

Bu_431	CACTTGAACCTCCCTTCTA G	FigS4	orf19.7127 RT-qPCR
Bu_432	CTTGGACATGAACAACAT ACTTG	FigS4	orf19.4054 RT-qPCR
Bu_433	GTTGTAGAGTCGACTGAC TCAAG	FigS4	orf19. 4054 RT-qPCR
Bu_434	TGTCTGACCATGGGTATA CCA	Fig2D	*Novel_ChrR_R093* RT-qPCR
Bu_435	CCGTAGCCCTAACCCTAA TT	Fig2D	*Novel_ChrR_R093* RT-qPCR
Bu_436	GACGCTAGAAGCTTGGTG TC	FigS4	orf19.2160 RT-qPCR
Bu_437	CGTAAACCAGATTCCAGG TC	FigS4	orf19.2160 RT-qPCR
Bu_438	AAATACGAGGGGACCAGA AG	FigS4	orf19.7545 RT-qPCR
Bu_439	CTTCGATGTGGTGATTGC AC	FigS4	orf19.7545 RT-qPCR
Bu_440	CAGATGAAGAATGCAGTT GG	FigS4	*Novel_Chr3_001* RT-qPCR
Bu_441	TCTCCAGCACTGTTCACT CC	FigS4	*Novel_Chr3_001* RT-qPCR

Supplementary table 4. Plasmids used in this study

**Plasmid**	**Description**
pGEMURA3	*URA3* integration products (Wilson et al, 1999)
pGEMHIS1	*HIS1* substitution products (Wilson et al, 1999)
pRS-Arg4SpeI	*Arg4* substitution products (Wilson et al, 1999)
pHA_NAT	*NAT* substitution products (Gerami-Nejad et al, 2012)

Supplementary table 5. Gene expression profile of MRS-associated genes in sir2 Δ/Δ versus wild-type isolates

**Orf name**	**Gene name**	**FPKM ratio**	**Orf**	**Chr**	Distance from MRS
orf19.1233	*ADE4*	0.123204	Orf L	1	2 kb
orf19.4712	*FGR6-3*	0.466022	Orf C	1	Inside MRS
orf19.4713	-	0.0576256	Orf R	1	1 kb
orf19.1742	*HEM3*	-0.0372117	Orf L	2	4 kb
orf19.3490	*FGR6-4*	0.0658554	Orf C	2	Inside MRS

orf19.5316	*FGR29*	1.31931	Orf R	2	1 kb
orf19.1801	*CBR1*	-0.202934	Orf L	4	6 kb
orf19.1234	*FGR6-10*	0.349738	Orf C	4	Inside MRS
orf19.1235	*HOM3*	0.273063	Orf R	4	1 kb
orf19.4349	-	0.307224	Orf L	5	6 kb
orf19.2655	*BUB3*	-0.454529	Orf R	5	1 kb
orf19.5773	-	-0.899313	Orf L	6	3 kb
NOVEL-Ca21chr6-037	-	-0.571963	Orf C	6	Inside MRS
orf19.1221	*ALG2*	0.0469119	Orf R	6	1 kb
orf19.7006	-	0.109973	Orf L	7	5 kb
NOVEL-Ca21chr7-016	-	0.228365	Orf C	7	Inside MRS
orf19.6898.1	-	0.122529	Orf R	7	3 kb
orf19.3695	-	-0.166999	Orf L	7	3 kb
orf19.5191	*FGR6-1*	0.795988	Orf C	7	Inside MRS
NOVEL-Ca21chr7-042	-	1.52733	Orf R	7	3 kb
orf19.3888	*PGI1*	-1.28362	Orf L	R	3 kb
NOVEL-Ca21chrR-061	-	1.49026	Orf C	R	Inside MRS
orf19.726	*PPZ1*	-0.0324678	Orf R	R	4 kb

Supplementary Table 5.

List of the MRS-proximal transcripts analysed in this study. The Log FPKM ratio of transcripts detected in sir2 Δ/Δ versus wild-type isolates is indicated. Orf numbers, gene name, chromosomal position and distance of each transcripts to MRS repeats is indicated.

Supplementary table 6. Gene expression profile of subtelomeric genes in sir2 Δ/Δ versus wild-type isolates

**Gene**	**Gene name**	**FPKM ratio**	**Orf #**	**Chr**	Distance from Telomeric repeats
NOVEL_Ca21chr1-001	-	1.60282	Orf 1	1L	~2kb
NOVEL_Ca21chr1-002	-	2.75269	Orf 2	1L	~3kb
orf19.6115	-	3.76323	Orf 3	1L	~4kb
orf19.6114	-	1.98411	Orf4	1L	~4kb
NOVEL_Ca21chr1-003	-	1.94456	Orf5	1L	~5kb
orf19.7278	-	1.91179	Orf 1	1R	~700 bp
orf19.7276.1	*TLO4*	0.527333	Orf 2	1R	~2kb
orf19.7277	-	-0.0468087	Orf 3	1R	~7kb
orf19.7275	*FGR24*	0.127306	Orf4	1R	~7kb
orf19.7274	-	0.542814	Orf5	1R	~7kb
NOVEL_Ca21chr2-001	-	1.37521	Orf 1	2L	~900 bp
NOVEL_Ca21chr2-002	-	0.659394	Orf 2	2L	~3 kb
orf19.1925	*TLO5*	1.06682	Orf 3	2L	~5 kb
orf19.1923	*RRN3*	1.1434	Orf4	2L	~7 kb
orf19.1920	-	2.10163	Orf5	2L	~8kb
NOVEL_Ca21chr2-097	-	0.029059	Orf 1	2R	~600 bp
orf19.5370	-	-0.417262	Orf 2	2R	~5 kb
orf19.5369	-	0.802781	Orf 3	2R	~6 kb
orf19.5368	-	-0.471883	Orf4	2R	~8 kb
orf19.5365.1	-	-0.705217	Orf5	2R	~10 kb
NOVEL_Ca21chr3-001	-	1.93103	Orf 1	3L	~2 kb
NOVEL_Ca21chr3-002	-	2.58399	Orf 2	3L	~3 kb
orf19.5474	-	1.71582	Orf 3	3L	~5 kb
orf19.5469	-	1.69472	Orf4	3L	~8 kb
orf19.5468	-	2.11177	Orf5	3L	~13 kb
NOVEL_Ca21chr3-067	-	1.29229	Orf 1	3R	~700 bp
orf19.6192	-	1.59438	Orf 2	3R	~4 kb
NOVEL_Ca21chr3-065	-	1.12003	Orf 3	3R	~5 kb
orf19.6191	*TLO8*	2.4063	Orf4	3R	~12 kb
orf19.6190	*SRB1*	1.37969	Orf5	3R	~15 kb
orf19.362	*TLO9*	1.32941	Orf 1	4L	~2 kb
NOVEL_Ca21chr4-001	-	0.750471	Orf 2	4L	~2 kb

orf19.364	-	0.395101	Orf 3	4L	~4 kb
orf19.366	-	0.950516	Orf4	4L	~5 kb
orf19.367	*CNH1*	0.0784385	Orf5	4L	~7 kb
NOVEL_Ca21chr4-075	-	0.416886	Orf 1	4R	1 kb
NOVEL_Ca21chr4-074	-	0.596045	Orf 2	4R	5 kb
orf19.3070	-	0.0911503	Orf4	4R	6 kb
orf19.3076	-	0.475355	Orf 3	4R	7 kb
orf19.3077	*VID21*	0.933247	Orf5	4R	9 kb
orf19.5700	*TLO11*	1.33724	Orf 1	5L	2 kb
orf19.5698	-	0.941254	Orf 2	5L	5 kb
orf19.5693	-	0.994588	Orf4	5L	6 kb
orf19.5694	-	0.779541	Orf 3	5L	7 kb
orf19.5691	*CDC11*	0.910976	Orf5	5L	10 kb
orf19.4055	-	0.705101	Orf 1	5R	2 kb
orf19.4054	*CTA24*	1.08844	Orf 2	5R	9 kb
NOVEL_Ca21chr5-051	-	1.76277	Orf4	5R	10 kb
orf19.4051	*HTS1*	0.996183	Orf 3	5R	13 kb
orf19.4048	*DES1*	0.656469	Orf5	5R	13 kb
orf19.6338	-	0.781793	Orf 1	6L	4 kb
orf19.6337	*TLO13*	1.14565	Orf 2	6L	6 kb
orf19.6336	*PGA25*	-0.155507	Orf4	6L	10 kb
orf19.6329	-	1.31487	Orf 3	6L	11 kb
orf19.6328	-	0.902296	Orf5	6L	12 kb
NOVEL_Ca21chr6-044	-	1.10283	Orf 1	6R	2 kb
orf19.2163	-	0.377088	Orf 2	6R	5 kb
orf19.2160	*NAG4*	4.41018	Orf4	6R	8 kb
orf19.2158	*NAG3*	2.84948	Orf 3	6R	10 kb
orf19.2157	*DAC1*	1.86075	Orf5	6R	12 kb
orf19.7125	-	0.454194	Orf 1	7L	1 kb
orf19.7124	*RVS161*	0.0573432	Orf 2	7L	2 kb
orf19.7123	-	-0.142634	Orf4	7L	3 kb
orf19.7121	-	1.02638	Orf 3	7L	3 kb
orf19.7119	*RAD3*	0.133712	Orf5	7L	6 kb
orf19.7127	*TLO16*	2.32205	Orf 1	7R	6 kb
orf19.7127.1	-	2.20881	Orf 2	7R	8 kb
orf19.7128	*SYS1*	0.110454	Orf4	7R	9 kb
orf19.7130	-	1.41063	Orf 3	7R	9 kb
orf19.7131	-	0.956586	Orf5	7R	10 kb
orf19.7545	-	2.06856	Orf 1	RR	5 kb
NOVEL_Ca21chrR-001	-	-0.770767	Orf 2	RR	7 kb
orf19.7544	*TLO1*	1.81897	Orf4	RR	9 kb
orf19.7539.1	-	0.471387	Orf 3	RR	12 kb
orf19.7539	*INO2*	0.600065	Orf5	RR	14 kb

orf19.7680	*CTA26*	1.26797	Orf 1	RL	900 bp
orf19.7678	*ATP16*	1.3481	Orf 2	RL	2 kb
orf19.7676	*XYL2*	1.66989	Orf4	RL	3 kb
orf19.7675	-	0.766025	Orf 3	RL	3 kb
orf19.7673	-	0.485425	Orf5	RL	4 kb

Supplementary Table 6.

List of the subtelomeric transcripts analysed in this study. The Log FPKM ratio of transcripts detected in sir2 Δ/Δ versus wild-type isolates is indicated. Orf numbers, gene name, chromosomal position and distance of each transcripts to telomeric repeats is indicated.
